# Two-component T-cell immunotherapy enables antigen pre-targeting to reduce cytokine release without forfeiting efficacy

**DOI:** 10.1016/j.nano.2025.102825

**Published:** 2025-04-30

**Authors:** M. Tommy Gambles, Isaac Kendell, Jiahui Li, Kyle Spainhower, Douglas Sborov, Shawn Owen, Alex Stark, David Bearss, Jiyuan Yang, Jindřich Kopeček

**Affiliations:** a Center for Controlled Chemical Delivery, University of Utah, Salt Lake City, UT 84112, USA; b Department of Molecular Pharmaceutics, University of Utah, Salt Lake City, UT 84112, USA; c Department of Biomedical Engineering, University of Utah, Salt Lake City, UT 84112, USA; d Huntsman Cancer Institute, University of Utah, Salt Lake City, UT 84112, USA; e U2TAH Therapeutics Accelerator, University of Utah, Salt Lake City, UT 84112, USA

**Keywords:** Lymphoma, Blinatumomab, Biomolecular engineering, Nanoconjugates, Pretargeting, T cells, Immunotherapy

## Abstract

Contemporary T-cell immunotherapies, despite impressive targeting precision, are hindered by aberrant cytokine release and restrictive targeting stoichiometry. We introduce a two-component T-cell immunotherapy targeting B-cell malignancies: Multi-Antigen T-Cell Hybridizers (MATCH). This split antibody technology differs from current therapies by separating cancer cell-targeting components from T cell-engaging components. We demonstrate that this two-component structure facilitates tunable T-cell activation. αCD19 and αCD20 MATCH, administered in two steps, are both compared to the clinical standard bispecific antibody, blinatumomab. In vitro two-dimensional dose analysis and cytokine release data indicate MATCH improves cancer clearance with reduced cytokine release. Cytolytic mechanisms of action are evaluated. αCD20 MATCH anti-cancer efficacy is assayed using a human lymphoma murine model. Decreasing T-cell engager dose 10-fold yields comparable efficacy to non-reduced doses. Ultimately, this split-antibody paradigm may enhance antigen targeting while reducing cytokine release, with such safety and efficacy advantages augmented by the future possibility of multi-antigen targeting with MATCH.

## Introduction

T-cell immunotherapies have revolutionized the treatment of B-cell malignancies. Six Chimeric Antigen Receptor (CAR) T-cell therapies and 11 bispecific antibodies (BsAbs) have been approved by the Food and Drug Administration in the past decade to treat hematological cancers.^1^ CAR-T cell therapies and BsAb immunotherapies are currently approved as second-line options against hematological malignancies. BsAbs include heterodimerized immunoglobulins (IgG), IgG-appended constructs, or Fc-devoid, bifunctional fusion proteins, such as bispecific T-cell engagers (BiTEs).^2,3^ While T-cell immunotherapies continue to improve prognoses, they are not risk-free. Many such systems are stoichiometrically limited by molecularly fixed structures, combining effector cell-recruiting and target cell-engaging domains into an inseparable unit. Consequentially, effector function can only be attenuated at the cost of reduced antigen targeting, and repeated targeting risks excessive toxicity.

Despite promising efficacy, T-cell therapies are hindered by significant side-effects. T cell-related side-effects, including cytokine release syndrome (CRS), immune effector cell-associated neurotoxicity syndrome (ICANS), and prolonged hematologic toxicity prevent broad utilization in the clinic.^4^ Systemic hypersecretion of IFNγ and TNFα by activated T-cells ignites innate immune cells throughout the body to release a cascade of pro-inflammatory cytokines. The resulting spike in inflammatory response can lead to CRS.^5^ CRS occurs in upwards of 90 % of patients receiving CAR-T cell therapy^6^ or BsAb therapy.^7^ Aberrant cytokine release can initiate or contribute to ICANS.^8^ Severe ICANS occurs in 10–30 % of patients receiving CAR-T cell therapy^9,10^ and 5–20 % of patients receiving BsAb therapy.^11^ Many patients achieve remission after T-cell therapy treatment; however, relapse or refractory disease is frequent.^12^ Between 30 and 60 % of lymphoma patients relapse by six months after CAR-T cell therapy^13^ and most multiple myeloma patients relapse by year two.^14^

The risk-benefit ratio often restricts implementation of T-cell therapies due to toxicity.^15^ Currently, no BsAb-specific consensus guidelines for CRS management exist. Guidance is typically based on CAR-T cell therapy recommendations.^16^ The degree of CRS is milder with BsAb therapy than CAR-T therapy, with rare Grade III and lack of Grade IV events.^17^ Nevertheless, BsAbs are limited in their ability to activate naive T-cells, reducing the overall magnitude of cytokine production in comparison to CAR-T cells infusions. BsAbs have characteristically lower incidence and severity of CRS and ICANS, which allow them to be administered to a broader patient population^18^; however, lower T-cell activation generally correlates with lower response rates. For example, no curative evidence of αCD19 BsAbs as a second- or third-line therapy exists.^19^ The clinical success and utility of T-cell immunotherapies is robust and undeniable; however, the technologies are marred by the inability to balance T-cell activation with associated inflammation.

We propose a split-antibody T-cell immunotherapy which involves antigen pre-targeting, mitigating aberrant cytokine release and enabling patient-specific dosing for effector cell recruitment. Multi-Antigen T-Cell Hybridizers (MATCH) is a two-component T cell-recruiting platform technology. MATCH consists of a T-cell engaging αCD3 antibody binding fragment (Fab′) coupled with select B cell-directed Fab′s.^20^ The αCD3 Fab′ (Fab′_CD3_) is modified to recognize and self-assemble with one B cell-directed Fab′ (Fab′_CD19_, Fab′_CD20_, Fab′_BCMA_, Fab′_SLAMF7_, or Fab′_CD38_). This interaction between Fab′s is achieved by complementary morpholino oligonucleotide (MORF) strands. Congruent MORF strands are conjugated to each respective Fab′.^21–23^ MATCH hybridization of corresponding Fab′-MORF conjugates mediates T-cell recruitment and activation against malignant B-cells. Separation of the Fab′_CD3_-MORF dose facilitates tunable T-cell activation and, consequently, greater control over cytokine release and unwanted T-cell exhaustion.^24,25^ Self-assembled MATCH conjugates ultimately resemble BiTE constructs. However, the split-antibody functionality of MATCH allows independent dose optimization in both cancer cell targeting and T-cell recruitment; such personalization is unachievable with current BsAb formats.^26^ A technology capable of personalizing doses specific to a patient’s condition could greatly improve safety and efficacy.^27–30^ To demonstrate the flexibility of MATCH, we identified optimal dose ratios of B-cell engager-to-T-cell engager that significantly deviate from a one-to-one ratio. Specifically, we show a 10-fold decrease in T-cell engager significantly decreases cytokine release *in vitro* without sacrificing anti-cancer efficacy. The 10-fold reduction in T-cell engager dose also cleared 7/7 mice of human Burkitt’s lymphoma for 150 days, outperforming higher T-cell engager doses. These findings suggest a two-component T-cell immunotherapy enables tunability of T-cell activation which could reduce immunotherapy side-effects and enhance dosing flexibility.

## Methods

### Cell culture

Healthy donor, naïve T-cells were collected by negative isolation from donor PBMC samples purchased from StemCell Technologies (Vancouver, CAN). Freshly isolated primary T-cells were cultured in ImmunoCult^™^-XF T-Cell Expansion Medium supplemented with ImmunoCult^™^ Human CD3/CD28 T-Cell Activator (25 μL/mL) and IL-2 (600 IU/mL). Raji human Burkitt’s B-cell non-Hodgkin’s lymphoma cell line was acquired from American Type Culture Collection (ATCC). Raji cells were cultured in RPMI 1640 medium supplemented with fetal bovine serum (10 % v/v), penicillin (100 units mL^−1^), and streptomycin (0.1 mg/mL) at 37 °C in a 5 % CO_2_ humidified atmosphere.

### Synthesis of Fab′-MORF conjugates

Fab′-MORF conjugates were synthesized as previously described.^21–23^ Briefly, immunoglobulins of rituximab, αCD19 (SJ25-C1, IchorBio), and αCD3 (UCHT-1, IchorBio) were converted to Fab′ fragments by enzymatic digestion (pepsin) and reduction (TCEP). A single 25 base morpholino oligonucleotide strand (MORF1) was conjugated to the αCD20 and αCD19 Fab′ fragments by thiol-maleimide ‘click’ chemistry through a bifunctional polyethylene glycol linker (SMPEG_2_). A complementary, 25 base morpholino oligonucleotide strand (MORF2) was conjugated to the αCD3 Fab′ fragment using a SMPEG_2_ linker. Detailed synthesis and characterization procedures are presented in Supplementary Information.

### Cellular mechanisms of action

MATCH-induced perforin pore formation on target Raji cells was imaged by confocal microscopy using an anti-perforin antibody (dG9, BioLegend). Calcium influx was quantified by dosing cells in Ca^2+^ enhanced (2.5 nM) RPMI 1640 medium and measuring fluorescence signal of Fluo-3 AM calcium indicator (5 μM) after 1 h MATCH or blinatumomab treatment. Mitochondrial depolarization was quantified by flow cytometry (FACSCanto) and confocal microscopy after 2 h treatment using the mitochondrial membrane potential sensor, JC-1 (Invitrogen). Caspase 3 activity was quantified after 2 h treatment using the caspase 3 activation indicator, PhiPhiLux^®^-G_1_D_2_ (OncoImmunin), by flow cytometry (FACSCanto). Apoptosis induction was quantified after 4 h treatment using annexin V and propidium iodide staining by flow cytometry (FACSCanto). For the FasL blockade assessment, the cells were dosed in combination with an anti-FasL antibody. For the granzyme inhibition assessment, healthy donor, naïve T-cells were pre-treated with a protease inhibitor cocktail for 30 min before co-culture with Raji B-cells. Apoptotic cell counts in the FasL blockade and the granzyme inhibitor cohorts were normalized to the respective αCD20 MATCH or blinatumomab treatment (without inhibition) to quantify apoptosis inhibition for each component.

### In vitro comparison of premixed and consecutive administration

MATCH was assayed for T-cell activation against malignant B-cells (Raji, human Burkitt’s lymphoma) *in vitro* and *in vivo*. MATCH was compared to the clinical standard bispecific T-cell engager, blinatumomab (Blincyto^®^) for efficacy, cytokine release, cellular mechanisms of action, and *in vivo* anti-cancer efficacy. MATCH anti-cancer efficacy was evaluated as self-assembled, single dose (“premixed”) and as a separately dosed (“consecutive”) therapy. Two-dimensional dose analysis was performed using luciferase-expressing Raji B-cells co-cultured with healthy donor, naïve T-cells in a 1:1 ratio after 24 h in 96-well plates using a dose titration (100 nM, 50 nM, 10 nM, 5 nM, 1 nM, 500 pM, 100 pM, and 0 nM). Viable Raji cells were quantified using a Perkin Elmer IVIS system to detect bioluminescence after addition of D-luciferin (10 μL of 15 mg/mL stock) to each well.

### In vitro cytokine release evaluation

MATCH-induced *in vitro* T-cell cytokine release was quantified and compared to blinatumomab using a LEGENDplex^™^ Human CD8/NK Panel 13-plex (BioLegend) which measures 13 cytolytic and pro-inflammatory molecules (IL-2, IL-4, IL-6, IL-10, IL-17a, granzyme A, granzyme B, granulysin, perforin, Fas, FasL, IFNγ and TNFα). Raji and healthy T-cells were co-cultured in a 96-well plate at a 2:1 ratio for 24 h. Both αCD19 and αCD20 MATCH were assayed in both premixed and consecutively administered formulations at five concentrations (50 nM, 10 nM, 5 nM, 1 nM, and 500 pM). Cytokine release was quantified and compared using flow cytometry (FACSCanto).

### Combinatorial checkpoint inhibitor treatment

The enhancement of MATCH therapy with combinatorial antibody blockade therapies was evaluated *in vitro*. αCD19 and αCD20 MATCH (50 nM) anti-cancer efficacy was compared with and without the addition of PD-1, IL-10, or KLRG-1 antibodies (0.1, 1.0, or 10 μg mL^−1^). A co-culture of Raji B-cells and healthy T-cells at a 1:3 ratio was incubated with therapy for 48 h and evaluated for Raji cell depletion using flow cytometry (FACSCanto). The higher tumor cell burden was utilized to produce overactivated or exhausted T-cells.

### In vivo consecutive MATCH dosing - Fab′_CD3_-MORF2 dose optimization

A human xenograft NHL murine mouse model was devised such that T cell-to-cancer cell ratio was tightly controlled. Female, 8-week-old SCID C⋅B-17 mice were intravenously inoculated on Day 0 with a co-culture of healthy donor, naïve T-cells (2 × 10^6^) and Raji-(luciferase expressing) human Burkitt’s B-cell NHL cells (4 × 10^5^) in a 5:1 T cell-to-target cell ratio. Mice were randomly distributed into treatment cohorts. One-hour post-inoculation, consecutive αCD20 MATCH was administered. Briefly, mice were given two tail vein injections 5 h apart. The first injection contained the B-cell engager Fab′_CD20_-MORF1 (60 μg); the second injection contained the T-cell engager Fab′_CD3_-MORF2 at different doses for each treatment group (60 μg, 20 μg, 6 μg, or 2 μg). For control cohorts, blinatumomab (60 μg) or αCD20 premixed MATCH (60 μg) was administered *via* single tail vein injection. Time points for target cell inoculation and the administration of effector T-cells and MATCH conjugates were selected to facilitate tight control of effector-to-target ratio. Mice were monitored for 21 weeks for cancer progression by IVIS imaging through bioluminescence intensity (see Supplementary Fig. S19 for complete array of IVIS images). Body weights for each mouse were recorded regularly (Supplementary Fig. S20). Moribund mice demonstrating symptoms of advanced illness (hind-limb paralysis) were euthanized in line with humane protocol and guidelines. Mice were regularly monitored for behavioral or physiological indicators of treatment toxicity. Long-term surviving mice (150 days) were euthanized and evaluated for residual disease in bone marrow (hind femur), spleen, and lumbar lymph nodes (Supplementary Figs. S21–S27).

### Statistics

Statistical analyses were performed using Microsoft Excel and Prism. No samples were omitted from any statistical calculations. Sample groups were compared using one-way ANOVA followed by the Tukey test; *p* < 0.05 was considered statistically significant. Experiments were performed in triplicate (*n* = *3*) and replicated at least two times. Fluorescence intensity for flow cytometry assays was quantified using the geometric mean average function. Survival curves of the murine model were analyzed for statistical significance by one-way ANOVA and Mantel-Cox logrank test.

## Results

### Synthesis and characterization of MATCH conjugates

**Fig. 1.**
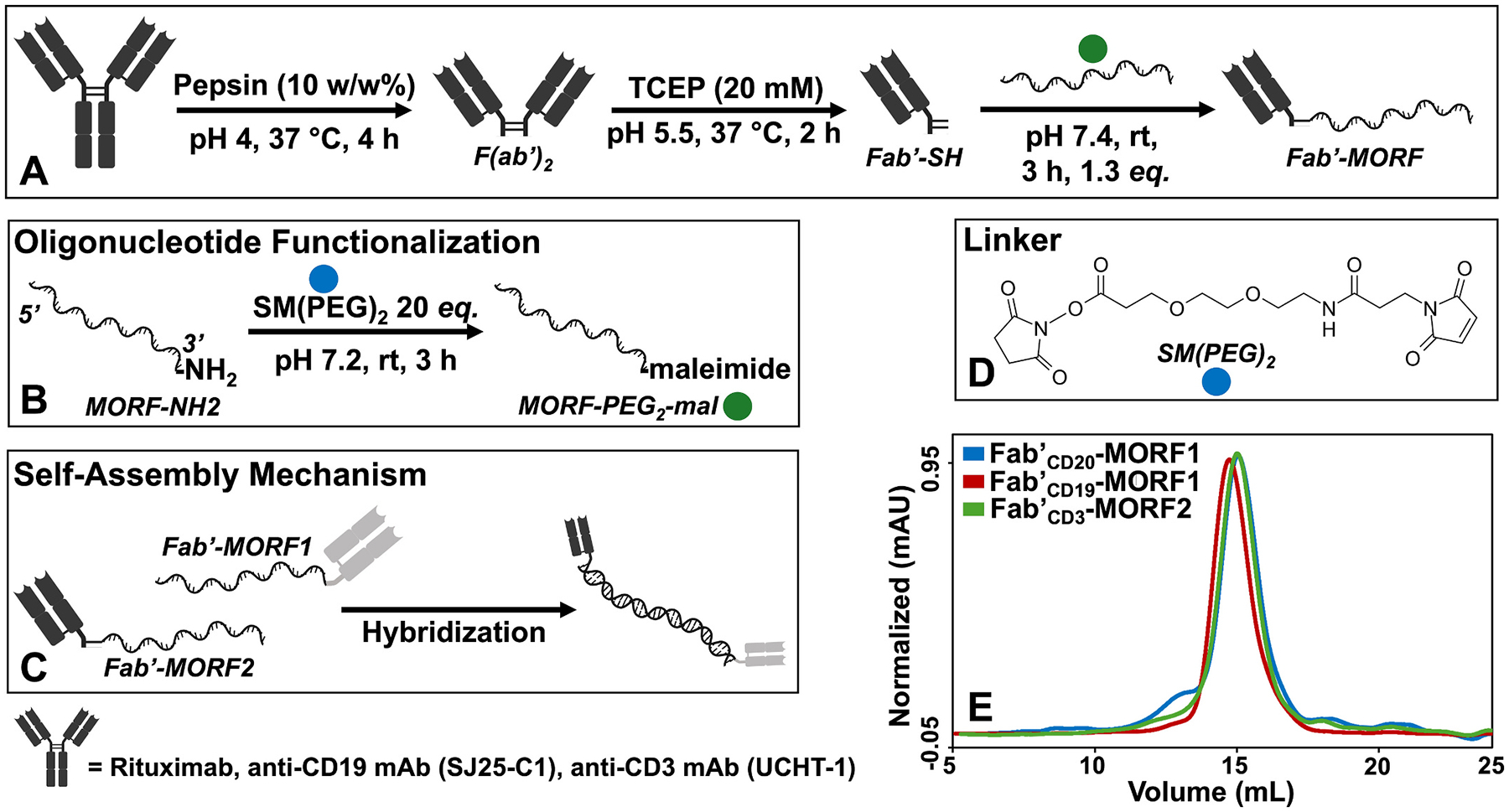
Synthetic schematic of MATCH nanoconjugates. (A) Synthetic scheme of generating Fab′-MORF conjugates from whole antibody starting material. (B) Linker coupling of *3*′-amine functionalized morpholino oligonucleotides using a bifunctional ethylene glycol dimer. (C) Hybridization of two complementary Fab′-MORF conjugates. Conventionally, Fab′-MORF1 conjugates are B-cell engagers and Fab′-MORF2 conjugates are T-cell engagers. (D) Chemical structure of bifunctional oligoethylene glycol (PEG) linker. (E) Size exclusion chromatogram overlay of Fab′_CD20_-MORF1 (blue), Fab′_CD19_-MORF1 (red), and Fab′_CD3_-MORF2 (green) products used in this study. Chromatographs were detected on a Superdex 200 10/300 GL column in PBS (pH 7.4) at 0.4 mL min^−1^.

Fab′-MORF conjugates are synthesized from whole antibody starting materials by 1) enzymatic cleavage, 2) disulfide bond reduction, and 3) thiol-maleimide-mediated linkage to the 25-base morpholino oligonucleotide (Fig. 1A–D).^23,31,32^ Optimizations in both polyethylene glycol (PEG) linker length, MORF base pair identity and oligomer length have been performed previously.^22^ Final products are characterized for purity, molecular weight, MORF-to-Fab′ substitution ratio, and dimerization efficiency.^33–37^ Each conjugate is subject to rigorous quality control (Supplementary Figs. S1 & S2), including purity, MORF covalent attachment, MORF/Fab′ substitution ratio, confirmation of complementation between MORF1 and MORF2 nanoconjugates, T-cell and target cell binding efficiency, and target cell depletion efficiency defined as killing of ≥90 % target cells after 24 h co-culture in a one-to-one T cell-to-target cell ratio.^19^ For this study, Fab′_CD19_-MORF1, Fab′_CD20_-MORF1, and Fab′_CD3_-MORF2 were newly synthesized and characterized according to standard protocol (Fig. 1E).

### MATCH treatment results in T cell-mediated cytotoxicity by perforin pore formation and granzyme caspase activation in target cancer cells

**Fig. 2.**
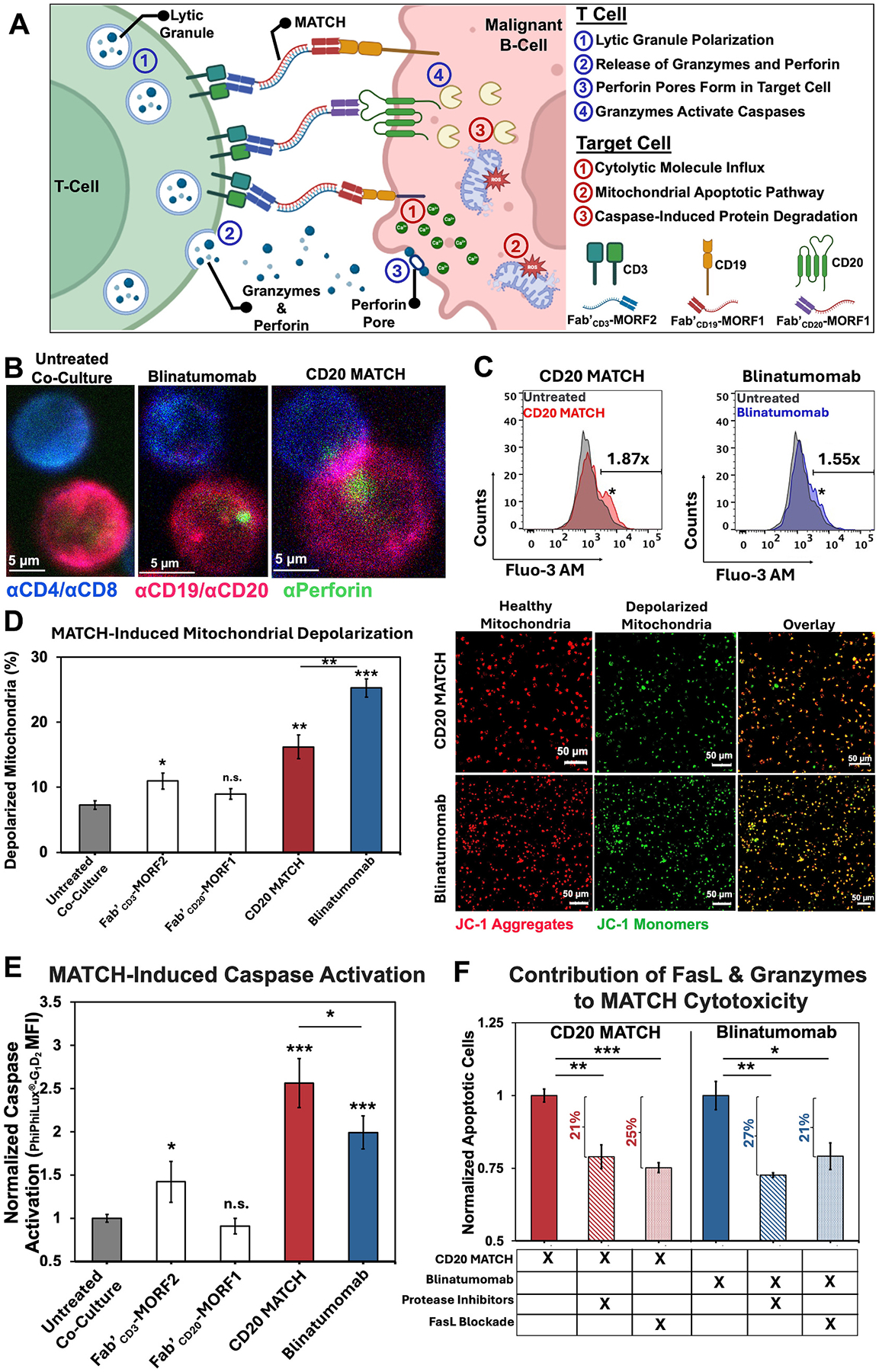
Mechanisms of αCD20 MATCH-induced cytotoxicity in Raji cells. (A) Schematic depiction of MATCH at the synapse of a malignant B-cell and an effector T-cell. Classical T cell-mediated cytotoxicity pathways were verified in response to MATCH treatment, with blinatumomab as positive control. (B) Live-cell confocal microscopy (Zeiss 700) of healthy human T-cells (blue) co-cultured with target Raji B-cells (pink) showing MATCH-induced perforin pore formation (green) in target cells after 1 h incubation. (C) Flow cytometry (FACSCanto^™^) quantification of MATCH-induced calcium influx in target cells after 1 h incubation with T-cells. Raji cells were pre-loaded with Fluo-3AM calcium indicator (5 μM). Fluorescence was normalized to untreated co-culture Raji cells (grey). (D) Flow cytometry quantification and live-cell confocal microscopy of MATCH-induced mitochondrial depolarization. Mitochondrial depolarization of individual Fab′-MORF conjugates was compared to MATCH using JC-1 dye (4 μM). Overlay of red (healthy mitochondria) with green (depolarized mitochondria) produces yellow. (E) MATCH-induced caspase-3 activation was quantified using a PhiPhiLux^®^-G_1_D_2_ kit (OncoImmunin). A co-culture of healthy T-cells and Raji B-cells was incubated for 3 h with a MATCH dose. Caspase activation was measured using flow cytometry and normalized to untreated co-culture MFI baseline. (F) Determining contribution of Fasligand and granzymes to overall MATCH-induced apoptosis of target Raji cells. Apoptosis was measured using annexin V/propidium iodide staining on flow cytometry. Inhibited T-cells were normalized to non-inhibited control. For FasL contribution, MATCH was administered in combination with an αFasL antibody (NOK-1; BioLegend). For granzyme contribution, MATCH was performed in combination with a protease inhibitor cocktail (Halt^™^). All experiments were performed in triplicate. ***p < 0.001, **p < 0.01, *p < 0.05, n.s. not significant by One-Way ANOVA and Tukey test.

We probed several T cell-mediated cytolytic mechanisms (Fig. 2A) within target Raji B-cells when incubated with αCD20 MATCH or the clinical standard BsAb blinatumomab (αCD3/αCD19) to verify that MATCH induces classic T cell-mediated cytotoxicity. Perforin pore formation was observed in target cell membranes (Fig. 2B) using anti-perforin immunostaining in both treatment groups (Supplementary Figs. S3–S5). Significantly higher cytosolic calcium was observed with both treatments (Fig. 2C). Occurrence of depolarized mitochondria increased in response to both αCD20 MATCH and blinatumomab, as confirmed by flow cytometry and confocal microscopy (Fig. 2D). Both treated cohorts of cells showed significantly higher levels of mitochondria depolarization than untreated control; however, blinatumomab induced even greater mitochondrial depolarization than MATCH. Both αCD20 MATCH- and blinatumomab-treated groups had significantly higher levels of caspase-3 activation over untreated cells (Fig. 2E), while αCD20 MATCH also had significantly higher caspase-3 levels than blinatumomab. Overall contributions to apoptosis from granzymes and death receptor ligand engagement (FasL) were quantified using inhibitors (Fig. 2F). Apoptotic Raji B-cell counts following treatment with therapy and each inhibitor were normalized to treatment with MATCH or blinatumomab alone. In both treatment cohorts, granzyme inhibition and FasL inhibition each decreased overall apoptotic efficacy of T-cells on Raji cells between 21 and 27 %, such that both pathways together accounted for about half of overall apoptosis.

### Comparison of premixed and consecutive MATCH identifies nonstandard optimal dose ratios

The two-component nature of MATCH allows two administration methods. Premixture of complementary Fab′-MORF conjugates leads to hybridization and self-assembly before administration to cells (hereafter “premixed”). Consecutive administration of MATCH conjugates is a two-step process wherein the B-cell engager is administered first, followed by the T-cell engager (hereafter “consecutive”). For *in vitro* consecutive administration, cells are treated with the B-cell engager for 1 h at 37 °C. After 1 h, cells are collected, washed with PBS and resuspended in fresh RPMI 1640 medium with the T-cell engager dose for desired duration. *In vivo*, animals are dosed with B-cell engager (0 h) followed by the T-cell engager (5 h). Plasma half-life of Fab′-MORF conjugates facilitates plasma clearance of B-cell engagers before T-cell engager administration,^27^ such that unbound Fab′_B-cell_-MORF1 is cleared from the blood before Fab′_CD3_-MORF2 administration.

**Fig. 3.**
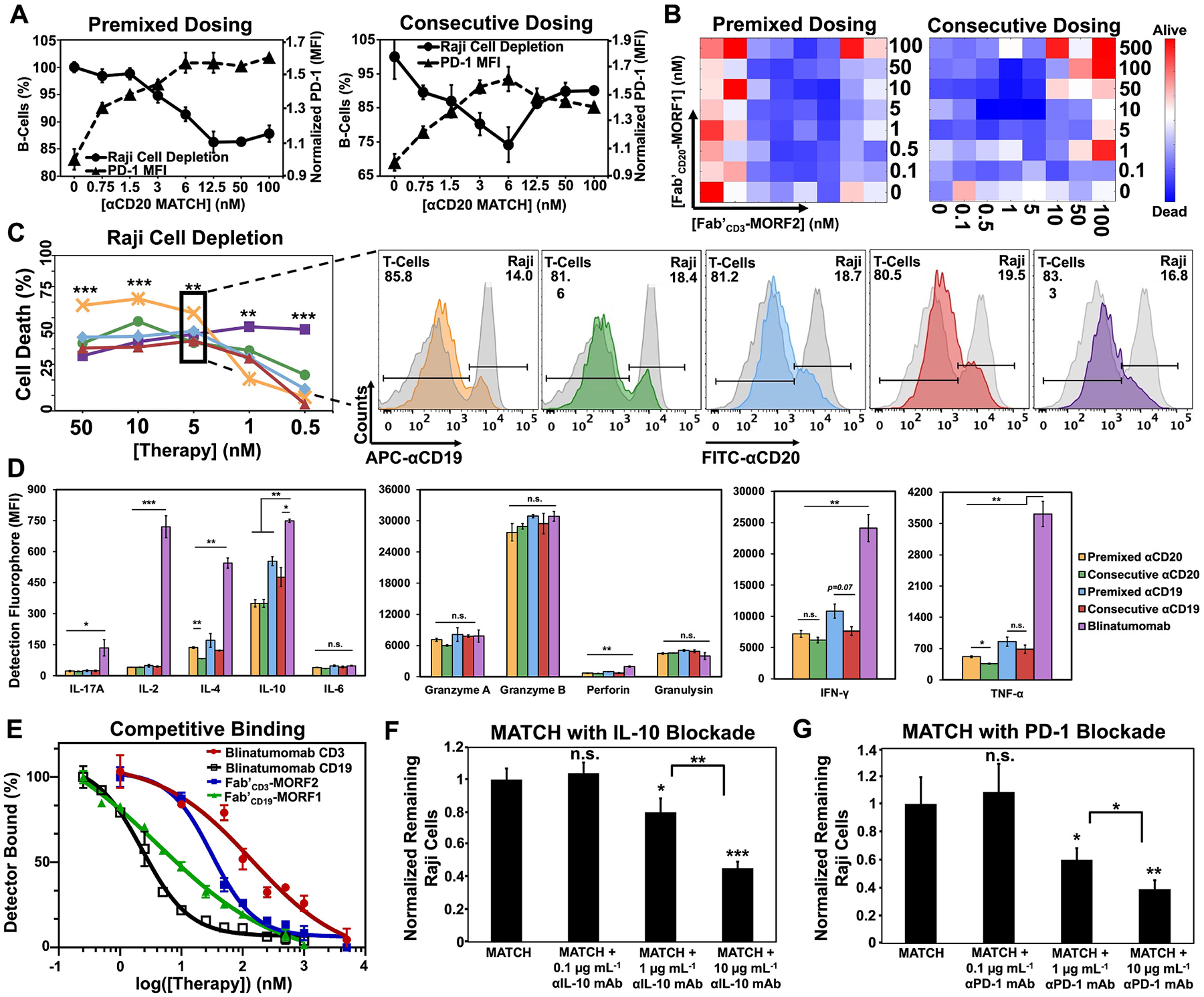
*In vitro* comparison of premixed and consecutive MATCH. (A) Raji B-cell depletion after 24 h incubation with either premixed αCD20 MATCH conjugates (left) or consecutive αCD20 MATCH (right). The T cell-to-Raji cell ratio was 1:3. Depletion of Raji cells and T-cell PD-1 expression was measured by flow cytometry (FACSCanto). (B) Two-dimensional dose heat maps were generated using luciferase-expressing Raji cells co-cultured with T-cells in a 1:1 ratio for 24 h. Raji cell death was quantified by luciferin addition and subsequent bioluminescence imaging (bioluminescence indicating residual target cells). (C) *In vitro* target cell death for titrations of each MATCH formulation is compared to blinatumomab (line graph) and example residual cell histograms are presented for 5 nM treatments. (D) *In vitro* T-cell cytokine release comparison of αCD19 and αCD20 MATCH to blinatumomab was performed using a T/NK cell cytokine multiplex panel, with 5 nM dose presented here. Additional data can be found in Supplementary Figs. S5–S7. (E) Competitive binding assays were performed by flow cytometry on Fab′_CD3_-MORF2, Fab′_CD19_-MORF1, and blinatumomab for comparison. (F) Premixed αCD20 MATCH target cell depletion in combination with an αIL-10 antibody. (G) Premixed αCD20 MATCH target cell depletion in combination with an αPD-1 antibody. All experiments were performed in triplicate. ***p < 0.001, **p < 0.01, *p < 0.05, n.s. not significant by One-Way ANOVA and Tukey test.

We compared *in vitro* premixed MATCH and consecutive MATCH administration in-depth, using blinatumomab as the control. We postulated that consecutive administration would yield optimal dose ratios which deviated from a 1:1 ratio. First, target cell depletion assays compared dose-response relationships in premixed MATCH and consecutive MATCH (Fig. 3A). Raji B-cells were co-cultured with healthy donor, naïve T-cells (3:1 ratio and 24 h incubation) with αCD19 or αCD20 MATCH administered either as premixed or consecutive treatments. B-cell depletion by both premixed MATCH formulations (αCD19 and αCD20) followed a trend of increased depletion with increased dose before reaching a plateau (the maximum T-cell cytolytic activity in this assay). Interestingly, consecutive MATCH treatment yielded a different trend. The optimal anti-tumor Fab′_CD3_-MORF2 dose was observed at 6 nM and greater T-cell engager doses reduced efficacy. αCD19 MATCH and blinatumomab trends are presented in Supplementary Fig. S9.

These findings were expanded using two-dimensional dose analysis (Fig. 3B, original images are in Supplementary Fig. S10). Luciferase-expressing Raji B-cells were co-cultured with healthy donor, naïve T-cells. Biaxial titrations of both Fab′_CD20_-MORF1 and Fab′_CD3_-MORF2 doses were administered to examine the impact of non-equivalent dosing (for both premixed and consecutive MATCH). Intriguingly, the most effective doses for premixed MATCH and consecutive MATCH did not overlap. The maximum efficacy of premixed MATCH tended to follow a one-to-one ratio of [Fab′-MORF1]-to-[Fab′-MORF2] with maximum efficacy at 10 nM of each conjugate, consistent with the depletion assay plateau concentration. Alternatively, the maximum efficacy of consecutively dosed MATCH centered around a [Fab′-MORF1]-to-[Fab′-MORF2] ratio of 10:1, again supporting depletion assay observations. Higher doses of either Fab′_CD20_-MORF1 or Fab′_CD3_-MORF2 tended to decrease anti-cancer efficacy.

Based on this two-dimensional dose analysis, we hypothesized that dose reduction in consecutive MATCH produced lower overall cytokine release than premixed MATCH or blinatumomab. Pro-inflammatory T-cell cytokine concentrations were measured *in vitro* at five doses for each MATCH formulation (compared to blinatumomab), and B-cell depletion was also quantified by flow cytometry (Fig. 3C). At higher doses (5, 10, and 50 nM), both premixed and consecutive αCD19 and αCD20 MATCH therapy depleted comparable levels of Raji B-cells to blinatumomab. Premixed αCD20 MATCH repeated the previous dose response trend with a depletion plateau beginning around 10 nM. Premixed αCD20 MATCH depleted significantly more B-cells than any other therapy 5, 10 and 50 nM doses. Consecutive αCD20 MATCH also repeated previously observed trends with maximum depletion at the 10 nM dose and less efficacy at the 50 nM dose. Blinatumomab maintained T-cell cytolytic activity at all concentrations tested and produced significantly more B-cell depletion at lower doses (1 nM and 0.5 nM).

Fig. 3D highlights the cytokine release at 5 nM dose concentration (see also Supplementary Figs. S11–S13). Both premixed and consecutive MATCH resulted in significantly lower IL-17A, IL-2, IL-4, IL-10, IFNγ and TNFα levels than blinatumomab at all concentrations assayed. Additionally, the cytolytic molecules granzyme A, granzyme B, and granulysin were equivalent between MATCH and blinatumomab at 5, 10 and 50 nM doses. Equivalent cytolytic molecule levels explain the comparable B-cell depletion; however, the differences in interleukins, IFNγ, and TNFα suggest MATCH greatly reduces non-specific T-cell cytokine release. A combination of antigen affinity and binding epitope differences likely resulted in observed cytokine release disparity.^38^ Competitive binding assays showed blinatumomab (140 nM) was a slightly weaker binder to CD3 than Fab′_CD3_-MORF2 (31 nM) but a slightly stronger binder to CD19 (2.3 nM) than Fab′_CD19_-MORF1 (4.1 nM) (Fig. 3E). Further, consecutive αCD20 MATCH treatment significantly reduced cytokine levels for certain analytes (IL-4 and TNFα) in comparison to premixed αCD20 MATCH. A preliminary *in vivo* cytokine release study confirmed that at 2 h, consecutive administration αCD19 MATCH with a 10-fold reduction in the T-cell engager resulted in slightly lower mean plasma concentrations of IL-2, IL-4, IL-10, IL-17A, TNFα, granzyme A, granzyme B, granulysin, IFN-γ, perforin, and sFasL than all other treatment groups (see Supplementary Fig. S14).

The elevated anti-inflammatory cytokine (IL-10) levels observed in this study prompted us to investigate combinatorial therapies to maximize T-cell cytotoxicity against cancer cells by simultaneously blocking anti-inflammatory signaling. Premixed αCD20 MATCH was administered in combination with inhibitory antibodies to block T-cell suppression signaling. IL-10 blockade (Fig. 3F), and PD-1 blockade (Fig. 3G), administered in combination with MATCH significantly increased T cell-mediated Raji cell depletion. MATCH administered with either 1 μg mL^−1^ or 10 μg mL^−1^ doses of αIL-10 or αPD-1 antibodies killed significantly more Raji cells than MATCH alone. The 10 μg mL^−1^ combination dose induced T-cells to destroy over 2-fold more Raji cells (Supplementary Figs. S16–S18).

### Reduction of T-cell engager dose in vivo retains anti-cancer efficacy

**Fig. 4.**
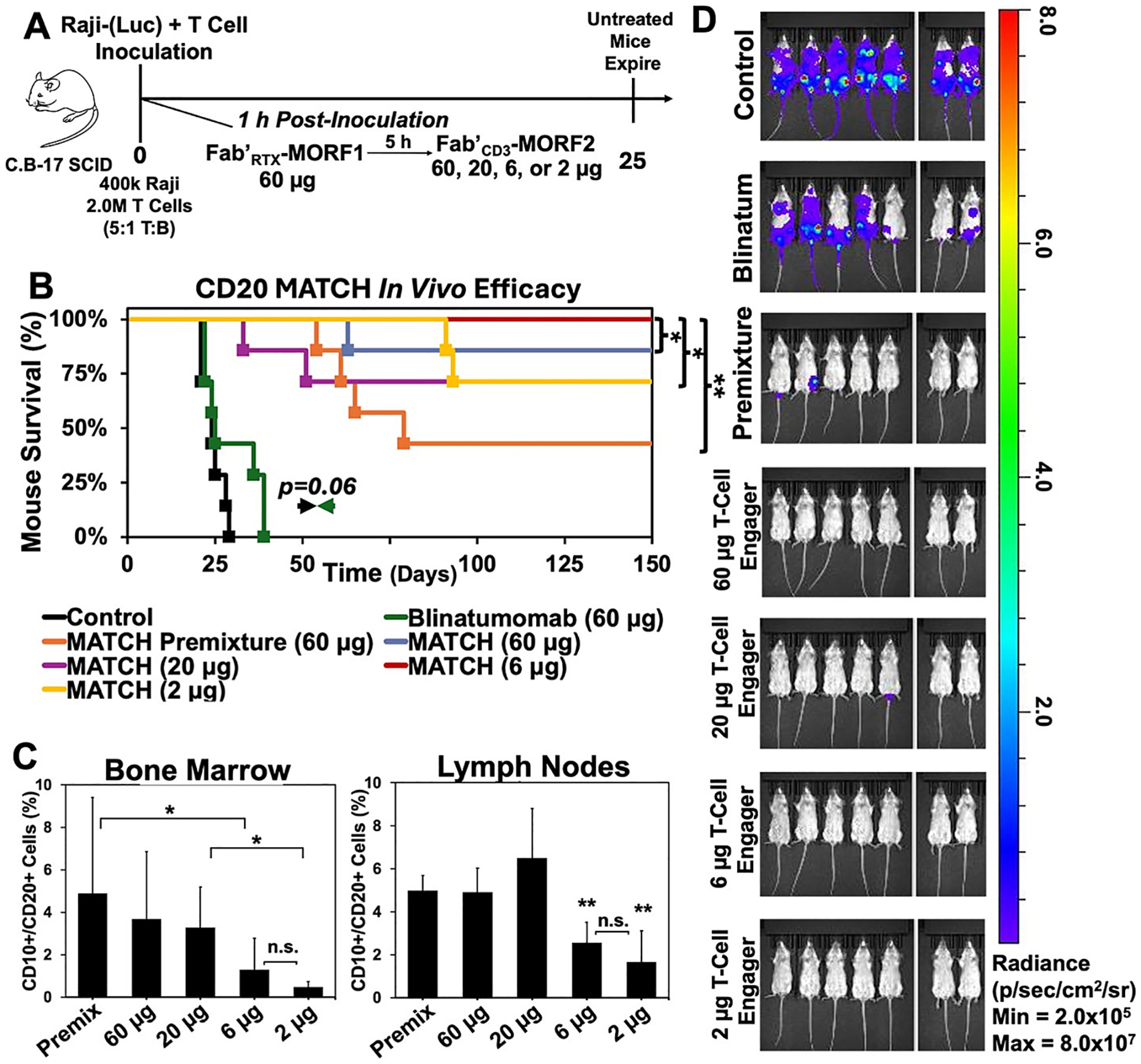
αCD20 MATCH *in vivo* efficacy against a human non-Hodgkin’s lymphoma xenograft in C.B-17 SCID mice. (A) Dosing regimen. A co-culture of luciferase-expressing Raji cells (4 × 10^5^) and healthy donor, naïve T-cells (2 × 10^6^) was inoculated *via* tail vein into female C.B-17 SCID mice. Cells were allowed to disseminate for 1 h before dosing began. Consecutive αCD20 MATCH was administered *via* tail vein in two consecutive injections 5 h apart. The first injection contained the B-cell engager, Fab′_CD20_-MORF1 (60 μg; 100 μL). The second injection contained the T-cell engager, Fab′_CD3_-MORF2, at serial dilutions for each cohort: 60 μg, 20 μg, 6 μg, or 2 μg. Consecutively dosed mice were also compared to a premixed αCD20 MATCH cohort who received a single MATCH injection containing the B-cell engager and the T-cell engager pre-hybridized (60 μg; 100 μL). Blinatumomab (αCD3/CD19) was administered as a single bolus tail vein injection (60 μg; 100 μL) as the clinical standard control treatment. (B) Paralysis-free survival curves plotting mouse survival up to 150 days post-inoculation. (C) Tissue analysis for residual disease in long-term surviving mice. Mechanically sieved cell suspensions were processed and analyzed for residual human B-cell presence *via* flow cytometry (FACSCanto). (D) Mice were monitored biweekly *via* bioluminescence detection using IVIS optical imaging. Sample images depicted were taken 21 days post-inoculation (additional images can be found in Supplementary Fig. S14). Images are adjusted to the luminescence scale depicted on the right in Radiance (p sec^−1^ cm^2 −1^ sr^−1^). Cohorts of mice (*n* = *7* per group) were randomly distributed (*α* = 0.05, power = 0.80); significance determined by logrank (Mantel-Cox) test. **p < 0.01, *p < 0.05, n.s. not significant by One-Way ANOVA and Tukey test.

We hypothesized an optimal T-cell engager to B-cell engager dose ratio below one-to-one would increase anti-cancer efficacy *in vivo*. We designed an *in vivo* model specifically to ascertain optimal dose concentration of the T-cell engager, Fab′_CD3_-MORF2. A human xenograft lymphoma mouse model was devised to tightly control T cell-to-cancer cell ratio (Fig. 4A). Female, SCID C⋅B-17 (Prkdc^−/−^) mice were intravenously inoculated on Day 0 with a co-culture of healthy donor, naïve T-cells and Raji (luciferase-expressing) human Burkitt’s B-cell lymphoma cells in a 5:1 T cell-to-target cell ratio.^20^ One-hour post-inoculation, consecutive αCD20 MATCH was administered. Mice were given two tail vein injections 5 h apart. The first injection contained the B-cell engager Fab′_CD20_-MORF1 (60 μg); the second injection contained the T-cell engager Fab′_CD3_-MORF2 at different doses for different treatment groups of mice (60 μg, 20 μg, 6 μg, and 2 μg). For control cohorts, blinatumomab (60 μg) and premixed αCD20 MATCH (60 μg) were administered *via* tail vein injection. Initiation of treatment on Day 0 is a viable xenograft lymphoma dosing schedule for immunocompromised mice that allows control over target cell-effector cell ratio.^39,40^ Mice were monitored for 21 weeks for cancer progression by IVIS imaging through bioluminescence intensity (Supplementary Fig. S19).

All untreated mice presented with significant bioluminescent signal by Day 21 post-inoculation and the average survival was 24.6 ± 3.1 days (Fig. 4B & D). The blinatumomab cohort also demonstrated significant bioluminescence signal at Day 21 post-inoculation with average survival of 31.1 ± 8.1 days. Blinatumomab-treated mice had significantly lower survival than any MATCH-treated cohort. We attribute the shorter survival to low half-life and rapid renal clearance of blinatumomab, not allowing sufficient retention and exposure to effectively recruit T-cells against cancer cells. All MATCH-treated cohorts contained long-term surviving mice; however, the premixed MATCH-treated cohort observed 4/7 mice succumbing to disease on average of 64.8 ± 10.5 days post-inoculation. Additionally, consecutive MATCH-treated cohorts of each T-cell engager dose observed imperfect survival except the 6 μg dose cohort. Interestingly, the B-cell depletion and two-dimensional dose analysis predicted an optimal T-cell engager dose ~10-fold lower than the B-cell engaging dose. Although the *in vivo* assessment observed widescale survival in all MATCH treatment groups, the only cohort with 7/7 mice surviving to experiment endpoint was the 10-fold T-cell engager dilution (6 μg dose) cohort.

Tissue was collected from each long-term surviving mouse and analyzed for residual human B-cells by flow cytometry (Fig. 4C). Residual disease (defined as ≥1 % total CD10^+^/CD20^+^ cells in sample) was observed in the bone marrow of 2 of 3 premixed cohort mice, 5 of 6 60 μg cohort mice, 4 of 5 20 μg cohort mice, 2 of 7 6 μg cohort mice, and 0 of 5 2 μg cohort mice. A similar trend was observed in minced lymph node tissue. The 6 μg and 2 μg cohorts had significantly fewer residual Raji cells than premixed, 60 μg, and 20 μg cohorts. The entire analysis is found in Supplementary Figs. S19–S27.

## Discussion

Pre-targeting in immuno-oncology decouples cancer cell targeting from the cytotoxic agent by administering the targeting component and the cytolytic component separately.^41^ Strategies incorporating pre-targeting follow three steps: i) administration of the cancer targeting motif, designed to bind to target antigens and eventually interact with the cytolytic molecule, ii) accumulation at target sites while free motif is cleared from the blood, and iii) administration of the complementary cytotoxic molecule, which engages with the bound targeting motif, resulting in target cell death. The pre-targeting recognition mechanism between components must occur bioorthogonally *in vivo*.^42,43^ Recent examples of pre-targeting approaches in the literature include a BsAb αCD20/αPEG scFv fusion protein to direct PEGylated liposomal doxorubicin to target B-cells,^44^ a streptavidin-functionalized drug-loaded nanoparticle that interacts with a biotinylated αVEGFR2 antibody to treat breast cancer,^45^ and two-component, receptor crosslinking technology that induces malignant B-cell death by clustering CD20 or CD38 on the cancer cell surface mediated by coiled-coil heterodimer formation or morpholino oligonucleotide hybridization.^46,47^ The authors named the latter technology Drug-Free Macromolecular Therapeutics (DFMT). DFMT has been successful at killing malignant B-cells *in vitro* and *in vivo*.^27,32–37,48^

MATCH recruits and activates T-cells in a pre-targeted approach using complementary oligonucleotides as the recognition mechanism between cancer cell engagers and a T-cell engagers. The non-therapeutic cancer cell-engager can be administered at high concentrations to coat the surfaces of target cells. After clearance of the cancer cell-engager from the blood, the therapeutic T cell-engager can be administered separately and at a desired dose distinct from the cancer cell-engager dose. Traditional T cell-engaging BsAbs are dose-limited by the onset of CRS due to both the cancer cell-engager and the T cell-engager being covalently bound within the same molecule.^49^ Decoupling the target cell engager from the T cell-engager in MATCH’s design allows for independent dosing and may lessen CRS-related dosing limitations.

Attenuated T-cell activation is only possible advantage of MATCH’s split antibody design. While this study examined single-target MATCH formulations, the MORF-mediated hybridization design enables and implies multi-antigen targeting in future studies. Further, the observation that a 10-fold reduction in T-cell engager dose improved treatment efficacy (as determined by cohort survival) strongly suggests that conjugate stoichiometry plays a meaningful role in therapeutic outcomes. If this consideration impacts immunotherapies broadly, then MATCH’s split-antibody design facilitates dose optimization that is not feasible with other (stoichiometrically limited) constructs, such as BsAbs, BiTEs, and other antibody-derived therapies. Additionally, separating cancer cell-engagers from effector cell-engagers makes supplementary dosing of a single conjugate possible without a corresponding increase in other conjugates. Theoretically, this could enable more patient-responsive immunotherapy dosing regimens than are currently available. As a result, further optimization of Fab′-MORF dosing based on varying patient parameters, such as antigen expression and cell counts, is required for effective translation into clinical use.

Both *in vitro* and *in vivo* data support the hypothesis that a personalized T-cell engager dose, independent of the B-cell engager dose, produces more anti-cancer efficacy and less aberrant T-cell cytokine release. In this study, we demonstrate that MATCH induces lower overall cytokine release *in vitro* than the control therapy (blinatumomab). Further, MATCH’s increased relative efficacy *in vivo* improved cancer clearance at the same or lower doses than the control therapy. Further studies will examine if MATCH’s safety profile outperforms control therapies in multi-dose regimens mirroring clinical practice. Importantly, our results strongly indicate a 1:1 ratio of T cell-engager-to-B cell-engager is not a universally optimal ratio. Varying tumor burden, T-cell counts, and target antigen expression levels between patients call for a more personalized approach to T-cell immunotherapy. MATCH enables responsive antigen specificity by molecular interchangeability of B-cell engagers. At the same time, MATCH facilitates independent T cell-engager dosing based on patient-specific variables. These results suggest that MATCH could provide a safer, more versatile and efficacious alternative for treating B cell malignancies.

## Supplementary Material

1

## Data Availability

The datasets generated and analyzed during the current the study are available from the corresponding authors on reasonable request.

## Appendix A. Supplementary data

The Supplementary Information includes additional descriptions and results of laboratory tests performed to further support the findings presented in the main text. Additional controls, original flow cytometry data, original confocal microscopy images, and detailed *in vivo* measurements. Supplementary data to this article can be found online at https://doi.org/10.1016/j.nano.2025.102825.
