# Supplementary material for: Two-component T-cell immunotherapy enables antigen pre-targeting to reduce cytokine release without forfeiting efficacy
Source: Nanomedicine. Author manuscript; Available in PMC 2026 May 19. (PMC13185708; doi:10.1016/j.nano.2025.102825)
Supplement: 1 [file NIHMS2174989-supplement-1.pdf]

SUPPLEMENTARY INFORMATION

Two-Component T-Cell Immunotherapy Enables Antigen Pre-Targeting To Reduce Cytokine Release Without Forfeiting Efficacy

M. Tommy Gambles[1,2,*,†], Isaac Kendell[3,†], Jiahui Li[1,2], Kyle Spainhower[4], Douglas Sborov[4], Shawn Owen[2,3], Alex Stark[5], David Bearss[5], Jiyuan Yang[1,2,*], Jindřich Kopeček[1,2,3,*]

[1] Center for Controlled Chemical Delivery, University of Utah, Salt Lake City, UT 84112, USA
[2] Department of Molecular Pharmaceutics, University of Utah, Salt Lake City, UT 84112, USA
[3] Department of Biomedical Engineering, University of Utah, Salt Lake City, Utah 84112, USA
[4] Huntsman Cancer Institute, University of Utah, Salt Lake City, UT 84112, USA
[5] U2TAH Therapeutics Accelerator, University of Utah, Salt Lake City, UT 84112, USA

*To whom correspondence should be addressed:

J. Kopeček, University of Utah, Center for Controlled Chemical Delivery, 2030 East 20 South, Biopolymers Research Building, Room 205B, Salt Lake City, Utah 84112-9452, USA

Tel.: +1 (801) 581-7211

E-mail: jindrich.kopecek@utah.edu

J. Yang, University of Utah, Center for Controlled Chemical Delivery, 2030 East 20 South, Biopolymers Research Building, Room 205C, Salt Lake City, Utah 84112-9452, USA

Tel.: +1 (801) 581-7349

E-mail: jiyuan.yang@utah.edu

T. Gambles, University of Utah, L.S. Skaggs Pharmacy Research Institute, 30 South 2000 East, Skaggs Research Building, Room SKH 301, Salt Lake City, Utah 84112, USA

Tel.: +1 (801) 388-6703

E-mail: michael.gambles@utah.edu

†Authors contributed equally to this work.

For original data, please contact Jindřich Kopeček at jindrich.kopecek@utah.edu or Thomas Gambles at Michael.gambles@utah.edu.

Contents:

Detailed Materials and Methods

Figure S1: Synthesis characterization of Fab'-MORF MATCH conjugates.

Figure S2: Quality control of synthesized Fab'-MORF conjugates dimerizing via complementary MORF1-MORF2 hybridization.

Figure S3: Confocal microscopy of perforin pores – untreated co-culture control.

Figure S4: Confocal microscopy of perforin pores – blinatumomab.

Figure S5: Confocal microscopy of perforin pores – CD20 MATCH.

Figure S6: Flow cytometry gating of mitochondrial depolarization.

Figure S7: Overlayed flow cytometry histograms of caspase-3 activation.

Figure S8: Apoptosis quantification gating of MATCH with or without protease inhibitor or FasL blockade.

Figure S9: Consecutively dosed CD19-directed MATCH versus premixed dosed CD19-directed MATCH.

Figure S10: Original bioluminescence of luciferase-expressing Raji cells remaining in a 48-well plate after 24 h incubation with CD20-directed MATCH.

Figure S11: Full in vitro T-cell cytokine quantification data acquired using a multiplex kit.

Figure S12: Flow histograms of residual Raji B-cells after 24 h incubation with indicated therapy.

Figure S13: T-cell viability after 24 h incubation with indicated therapy.

Figure S14: *In vivo* plasma cytokine concentration following MATCH or blinatumomab treatment (2 h).

Figure S15: Competitive binding of MATCH conjugates on CD19 and CD3 compared to blinatumomab.

Figure S16: MATCH + PD-1 or IL-10 blockades flow cytometry histograms.

Figure S17: MATCH + KLRG-1 blockade normalized residual target cell counts.

Figure S18: T-cell rechallenge experiments measuring cell exhaustion (TIM-3, PD-1) and excitation (CD44).

Figure S19: Complete IVIS imaging for *in vivo* assessment of MATCH.

Figure S20: Individual mouse weights for *in vivo* assessment of MATCH.

Figure S21: *Ex vivo* residual Raji B-cells in mouse spleen for *in vivo* assessment of MATCH.

Figure S22: *Ex vivo* residual Raji B-cells for *in vivo* assessment of MATCH – group tissue comparison.

Figure S23: Flow cytometry residual disease quantification for premixed-treated cohort's long-term survivors.

Figure S24: Flow cytometry residual disease quantification for 60 μg T-cell engager cohort's long-term survivors.

Figure S25: Flow cytometry residual disease quantification for 20 μg T-cell engager cohort's long-term survivors.

Figure S26: Flow cytometry residual disease quantification for 6 μg T-cell engager cohort's long-term survivors.

Figure S27: Flow cytometry residual disease quantification for 2 μg T-cell engager cohort's long-term survivors.

DETAILED MATERIALS AND METHODS

The complementary phosphorodiamidate morpholino oligonucleotides were customized and purchased from Gene Tools (Philomath, OR, USA). Both strands were modified at the 3' terminus with a primary amine functional group. The specific sequences of each oligonucleotide are: "MORF1" 5'-GAGTAAGCCAAGGAGAATCAATATA-NH_2-3' and "MORF2" 5'-TATAATTGATTCTCCTTGGCTTACTC-NH_2-3'. Pepsin (porcine gastric mucosa) was purchased from Sigma Aldrich. Tris(2-carboxyethyl) phosphine (TCEP), the protease inhibitor cocktail (Halt^{TM}) and the bifunctional linker, succinimidyl-([*N*-maleidopropionamido]-diethyleneglycol) ester (SMPEG2), were purchased from Thermo Fisher Scientific (Rockford, IL, USA). *D*-luciferin was purchased from GoldBio (St. Louis, MO, USA). 4-(6-acetoxymethoxy-2,7-dichloro-3-oxo-9-xanthenyl)-4'-methyl-2,2'-(ethylenedioxy)dianiline-*N*,*N*,*N'*,*N'*-tetraacetic acid tetrakis (acetoxymethyl) ester (Fluo-3 AM), 5,5',6,6'-tetrachloro-1,1',3,3'-tetraethylbenzimidazoylcarbocyanine iodide (JC-1), and carbonyl cyanide 3-chlorophenylhydrazone (CCCP) were purchased from Invitrogen (Carlsbad, CA, USA). PhiPhiLux®G_1D_2 was purchased from OncoImmunin (Gaithersburg, MD, USA). The LEGENDplex^{TM} Human CD8/NK Panel 13-plex was purchased from BioLegend (San Diego, CA, USA). A list of commercially acquired antibodies is shown in Table 1.

Table 1. List of mAbs used for MATCH conjugate synthesis and for immunostaining of various *in vitro* procedures outlined in the methods.

Antibody	Clone	Fluorophore	Distributor
CD3	UCHT-1		IchorBio
CD19	SJ25-C1		IchorBio
CD20	Rituxan®		Huntsman Cancer Institute
Blinatumomab	AB_2910872		ThermoFisher
PD-1	A17188A	FITC	BioLegend
PD10	HI10a	PE	BioLegend
CD3	SK7	APC	BioLegend
CD19	HIB19	APC	BioLegend
CD20	2H7	APC	BioLegend
CD20	2H7	FITC	BioLegend
CD4	SK3	APC	BioLegend
CD8	SK1	APC	BioLegend
Perforin	dG9	FITC	BioLegend
IL-10	JES3-19F1		BioLegend
TIM-3	QA17A33		BioLegend
KLRG-1	14C2A07		BioLegend
FasL	NOK-1		BioLegend

Animals

All experiments involving animals were performed according to the protocol approved by the Institutional Animal Care and Use Committee (IACUC) and of the University of Utah. Female SCID C·B-17 mice (Prkdc$^{-/-}$) were purchased from Charles River Laboratories (Wilmington, MA, USA) and used for xenograft models once body weight reached 18 g. Mice were housed at constant room temperature (23 °C) and relative humidity (60 ± 5%) with free access to sterile water and food pellet, and a fixed 12 h light/dark cycle. For imaging, mice were anesthetized with 5% isoflurane and maintained under anesthesia with 1-2% isoflurane. Animals were

euthanized using carbon dioxide with a container fill rate of 50% of chamber volume displacement per minute. Following CO_2 asphyxiation, mice were cervically dislocated.

Synthesis of Fab'-MORF Conjugates

Briefly, whole antibody (5 mg, 5 mg mL^{-1}) was buffer exchanged into 100 mM citric acid buffer (pH 4.0) by centrifugal ultrafiltration (30 kDa MWCO membrane 3x volume washes) and enzymatically digested with pepsin (20 w/w%) for 16 h at 37 °C to generate the respective $F(ab')_2$ intermediate species. $F(ab')_2$ was purified and buffer exchanged into 100 mM citric acid buffer (pH 5.5) by centrifugal ultrafiltration (30 kDa MWCO membrane 8x volume washes). $F(ab')_2$ was reduced with TCEP (20 mM) for 3 h at 37 °C to generate the respective Fab' intermediate species. Fab' was purified, by centrifugal ultrafiltration (10 kDa MWCO membrane 8x volume washes), buffer exchanged into PBS pH 6.5 buffer, and used immediately. In parallel to the $F(ab')_2$ reduction reaction, 3' amine-functionalized MORF1 (or MORF2 for Fab'_{CD3}) was conjugated to the bifunctional $SM(PEG)_2$ linker by NHS-amine coupling. MORF (1.8 mg, 200 nmol) was dissolved in PBS pH 7.4 buffer (150 µL) and $SM(PEG)_2$ (4.25 mg, 10 µmol) was dissolved in DMSO (50 µL). The reaction was continued for 3 h at room temperature. The MORF-PEG_2-maleimide intermediate species was purified and buffer exchanged into PBS pH 6.5 buffer using centrifugal ultracentrifugation (3 kDa MWCO membrane, 10x volume washes). Pure MORF-PEG_2-maleimide (1.5 eq.) was added to pure Fab' (1 eq.) and reacted for 4 h at room temperature. The final produce, Fab'-MORF, was purified and buffer exchanged into PBS pH 7.4 buffer by centrifugal ultracentrifugation (30 kDa MWCO membrane, 10x volume washes).

Characterization and Quality Control of Fab'-MORF Conjugates

Each freshly prepared Fab'-MORF conjugate is characterized through quality control procedures including purity, MORF-to-Fab' substitution ratio, complementation of congruent MORF moieties, and T-cell activation efficacy. Purity is determined by FPLC using a Superdex 200 10/300 GL column in PBS pH 7.4 at 0.4 mL min^{-1}. The MORF-to-Fab' conjugation ratio is determined by protein concentration bicinchoninic acid (BCA) assay and MORF concentration is determined by UV-Vis (NanoDrop ND-1000 spectrophotometer) absorbance at λ=260 nm (ε_{MORF1} = 278,000 M^{-1} cm^{-1}; ε_{MORF2} = 252,120 M^{-1} cm^{-1}), and by mass spectroscopy (Xevo G2S Q-ToF). The ability of two complementary conjugates to self-assemble is assayed using one-to-one mixture of congruent Fab'-MORF species and analyzed by FPLC, dynamic light scattering, and by hypochromic effect on UV-Vis. Finally, a 24 h *in vitro* depletion assay of a target cancer cell co-cultured with healthy, naïve T-cells is dosed with the freshly manufactured conjugates to confirm cytolytic T-cell activation and subsequent B-cell depletion.

Premixed Versus Consecutive Dosing of Two-Component MATCH

The two-component nature of MATCH allows for two routes of dose administration. Premixture of complementary Fab'-MORF conjugates allows for hybridization before administration to cells. We refer to this administration as "premixed". In premixed administration, congruent $Fab'_{B\text{-}cell}$-MORF1 conjugate(s) are mixed with Fab'_{CD3}-MORF2 in a one-to-one MORF1-to-MORF2 ratio and dosed according to [MORF].

Consecutive dose administration of MATCH conjugates includes a two-step process in which the B-cell engager(s) are administered first, followed by the T-cell engager second. *In vitro*, cells are dosed with the B-cell engager(s) for 1 h at 37 °C. After 1 h, cells are collected, washed with PBS and resuspended in fresh RPMI 1640 medium with the T-cell engager dose for desired duration. *In vivo*, animals are dosed with B-cell engager(s) followed by the T-cell engager with a 5 h time lag between injections. Plasma half-life of Fab'-MORF conjugates allows for three half-lives to ensue before T-cell engager administration. Free, unbound $\text{Fab'}_{\text{B-cell}}$-MORF1 is cleared from the blood before Fab'_{CD3}-MORF2 administration.

Premixed Versus Consecutive Dosing Depletion Assay

Raji B-cells (1.5×10^5) and healthy donor, naïve T-cells (5×10^4) were co-cultured in 3-to-1 B cell-to-T cell ratio in a 48-well plate in 400 µL RPMI 1640 medium. Cells were incubated with or without titrated doses of CD19- or CD20-directed MATCH (100 nM, 50 nM, 25 nM, 12.5 nM, 6.1 nM, 3.0 nM, 1.5 nM, and 0.75 nM) administered either as a premixed or consecutive therapy. Therefore, premixed dosed MATCH kept the ratio of $\text{Fab'}_{\text{B-cell}}$-MORF1-to-$\text{Fab'}_{\text{CD3}}$-MORF2 constant at 1-to-1 for all doses. For consecutively dosed MATCH, the B-cell engager ($\text{Fab'}_{\text{CD19}}$-MORF1 or $\text{Fab'}_{\text{CD20}}$-MORF1) was administered at 100 nM for each treatment group for 1 h at 37 °C. Cells were collected, washed, and resuspended in fresh cell medium. The T-cell engager (Fab'_{CD3}-MORF2) was subsequently dosed at the concentrations identified above, effectively diluting the ratios of $\text{Fab'}_{\text{B-cell}}$-MORF1-to-$\text{Fab'}_{\text{CD3}}$-MORF2 1:1, 2:1, 4:1, 8:1, 16:1, 32:1, 64:1, and 128:1. Cells were incubated for 24 h at 37 °C. After 24 h, cells were collected, washed and immunostained with PE-αCD10, APC-αCD4/CD8, and FITC-αPD-1. Flow cytometry was used to quantify residual Raji cells present ($CD10^+/CD4^-/CD8^-$) and levels of PD-1 expression on T-cells ($CD4^+/CD8^+$; FITC geometric mean fluorescence intensity) as an indication of activation. Depletion assays were performed in triplicate and replicated two times. Data for CD19-directed MATCH can be found in Supplementary Figure S3.

Premixed Versus Consecutive Dosing 2-Dimensional Analysis Assay

Luciferase-expressing Raji B-cells (1×10^5) and healthy donor, naïve T-cells (1×10^5) were co-cultured in a 1-to-1 B cell-to-T cell ratio in a 48-well plate in 200 µL RPMI 1640 medium. Along the axes of the plates, $\text{Fab'}_{\text{CD20}}$-MORF1 and Fab'_{CD3}-MORF2 were dosed in serial dilution (100 nM, 50 nM, 10 nM, 5 nM, 1 nM, 500 pM, 100 pM, and 0 nM) creating a 2-dimensional matrix of 64 different dose combinations. Experiments were conducted for both premixed and consecutively dosed MATCH therapy. For consecutively dosed MATCH administration, Raji cells were treated with the identified concentrations of $\text{Fab'}_{\text{CD20}}$-MORF1 for 1 h at 37 °C followed by PBS wash and resuspension in fresh RPMI 1640 medium. Fab'_{CD3}-MORF2 was then administered at the corresponding concentration. Cells were incubated for 24 h at 37 °C. After 24 h, D-luciferin (10 µL of 15 mg/mL stock) was added to each well. The plate was immediately imaged with a Perkin Elmer IVIS system to detect bioluminescence signal. IVIS images were imported into Aura Imaging Software (Spectral Instruments). Region-of-interest grids were drawn across each image corresponding to the actual plate layout, isolating the signal form each well into a single grid element. Maximum radiance values for each grid element were exported into MATLAB into an array with the same spatial layout as the original plate. The maximum and minimum value of each array

were set as the heat scale maximum and minimum values, respectively. The array was converted into a two-dimensional grid with each array element assigned a color based on the relative value of that element with regards to the heat scale. The color scheme was set to red-blue (maximum-minimum) to aid visual interpretation (see Supplementary Code). Red indicates high signal, or presence of viable Raji B-cells. Blue indicates low signal, or lack of viable Raji B-cells.

In Vitro Multiplex Cytokine Release

The release of pro-inflammatory cytokines from activated T-cells was quantified at five doses (50 nM, 10 nM, 5 nM, 1 nM, and 500 pM) of CD19- and CD20-directed MATCH using a multiplex technology. The LEGENDplex^{TM} Human CD8/NK Panel 13-plex (BioLegend) measures 13 cytolytic and pro-inflammatory molecules (IL-2, IL-4, IL-6, IL-10, IL-17a, granzyme A, granzyme B, granulysin, perforin, Fas, FasL, IFNγ and TNFα) released upon effector cell activation. Premixed and consecutively dosed CD19- and CD20-directed MATCH therapies were compared to blinatumomab (αCD3/αCD19). Raji B-cells (5×10^4) and healthy donor, naïve T-cells (2.5×10^4) were co-cultured in a 2-to-1 B cell-to-T cell ratio in the multiplex 96-well plate in 200 µL RPMI 1640 medium. Dose administration for premixed and consecutively administered MATCH were conducted as described in the 2-dimensional analysis assay. At each therapy dose concentration the levels of cytokines, the depletion of Raji B-cells, and the depletion of T-cells were quantified. Main text highlights the results at 5 nM dose concentration; other dose information can be found in Supplementary Figures S5-S7. Each dose was performed in duplicate, and each experiment was replicated two times. Analysis was performed on a FACSCanto flow cytometer according to the LEGENDplex^{TM} instructions, including the creation of calibration curves using known concentrations of cytokines provided in the kit.

Competitive Binding Assay

Raji B-cells (2×10^5) or healthy donor, naïve T-cells (2×10^5) were added to a 48-well plate in 100 µL RPMI 1640 medium. Cells were cooled to 4 °C. Cells were then treated with consecutive dilutions of either Fab'$_{CD19}$-MORF1, Fab'$_{CD3}$-MORF2, or blinatumomab for 30 min at 4 °C. After 30 min, cells were collected, washed with PBS, and resuspended in 50 µL staining buffer containing either APC-αCD19 or APC-αCD3 (0.5 µg/well). Cells were immunostained for 30 min at 4 °C. After detection antibody treatment, cells were collected, washed with PBS, and analyzed for detection antibody binding using flow cytometry (FACSCanto). Mean fluorescent intensities were gathered for each dilution of therapy pre-treatment by measuring the fluorescence of the bound detection antibody. MFIs of detection antibodies were plotted and a non-linear regression curve fit was applied to compare binding constants as EC_{50} values. Non-linear least squares line fits were made using GraphPad Prism.

MATCH in Combination with PD-1, IL-10, TIM-3, or KLRG-1 Blockade

Antibody blockade experiments were performed by seeding healthy donor, naïve T-cells (5×10^4) and Raji cells (1.5×10^5) in a 24-well plate in 800 µL RPMI 1640 medium. Consecutively administered CD20-directed MATCH (50 nM) was administered to the cells. The respective blockade antibody (0.1, 1, or 10 µg mL^{-1}) was added at the time of Fab'$_{CD3}$-MORF2 administration. Cells were incubated for 48 h at 37 °C. After treatment, cells were

washed and immunostained with FITC-αPD-1, APC-αCD19, and PE-αCD10. The percentage of viable T- and B-cells was quantified by flow cytometry (FACSCanto) and normalized to the MATCH alone control to determine increase in Raji cell ($CD19^+$/$CD10^+$ population) death.

Confocal Microscopy of Perforin Pore Formation

Raji B-cells ($1x10^5$) and healthy donor, naïve T-cells ($1x10^5$) were co-cultured in a 1-to-1 B cell-to-T cell ratio in a 48-well plate in 400 µL RPMI 1640 medium. Cells were incubated with or without premixed CD20-directed MATCH (50 nM) or blinatumomab (50 nM) for 2 h at 37 °C. After 2 h, cells were collected, washed with PBS, immunostained with PE-αCD19 (for CD20 MATCH-treated), PE-αCD20 (for blinatumomab-treated), APC-αCD4/CD8, and FITC-αPerforin. Cells were immediately live-cell imaged using a confocal microscope (Zeiss 700). Original images can be found in Supplementary Figures S12-S14.

Calcium Influx Assay

Raji B-cells ($1x10^5$) were pre-treated with Fluo-3 AM calcium indicator (5 µM) in 400 µL RPMI 1640 medium for 30 min at 37 °C. Cells were washed and resuspended in fresh medium (supplemented with 2.5 nM Ca^{2+}) and T-cells. Cells were incubated with or without CD20-directed MATCH (50 nM) or blinatumomab (50 nM) for 1 h at 37 °C. After 1 h, cells were collected, washed, and immunostained with APC-αCD4/CD8 and PE-αCD10. Cells were washed and fluorescence of Fluo-3 AM indicator in Raji B-cells ($CD10^+$/$CD4^-$/$CD8^-$) was quantified using flow cytometry (FACSCanto).

Mitochondrial Depolarization Flow Cytometry

Raji B-cells ($1x10^5$) and healthy donor, naïve T-cells ($1x10^5$) in a 1-to-1 B cell-to-T cell ratio in a 48-well plate in 400 µL RPMI 1640 medium. Cells were incubated with or without CD20-directed MATCH (50 nM) or blinatumomab (50 nM) for 2 h at 37 °C. After 2h, the mitochondrial membrane potential sensor, JC-1, was added along with αCD4/CD8. For positive depolarized membrane control, a cohort of cells were treated with CCCP (0.5 µM) along with JC-1. Cells were incubated for an additional 30 min at 37 °C. After JC-1 treatment, cells were collected, washed and analyzed with flow cytometry (FACSCanto) for fluorescence of JC-1 aggregates (healthy mitochondria) and JC-1 monomers (depolarized mitochondria) in Raji B-cells ($CD4^-$/$CD8^-$). Experiment was performed in triplicate and replicated two times. Gating can be found in Supplementary Figure S15.

Mitochondrial Depolarization Confocal Microscopy

Raji B-cells ($1x10^5$) and healthy donor, naïve T-cells ($1x10^5$) were co-cultured in a 1-to-1 B cell-to-T cell ratio in a 48-well plate in 400 µL RPMI 1640 medium. Cells were incubated with or without CD20-directed MATCH (50 nM) or blinatumomab (50 nM) for 2 h at 37 °C. After 2 h, the mitochondrial membrane potential sensor, JC-1, was added. Cells were incubated for an additional 30 min at 37 °C. After JC-1 treatment, cells were collected, washed and immediately live-cell imaged using a confocal microscope (Zeiss 700). JC-1 aggregates (healthy mitochondria) are depicted as red and JC-1 monomers (depolarized mitochondria) are depicted as green. The

JC-1 aggregate and JC-1 monomer channels were overlayed and presented. More yellow/orange indicates more depolarized mitochondrial membranes in the sample.

Caspase Activation Assay

Raji B-cells ($1x10^5$) and healthy donor, naïve T-cells ($1x10^5$) were co-cultured in a 1-to-1 B cell-to-T cell ratio in a 48-well plate in 400 µL RPMI 1640 medium. Cells were incubated with or without CD20-directed MATCH (50 nM) or blinatumomab (50 nM) for 2 h at 37 °C. After 2 h, a caspase 3 activation indicator, PhiPhiLux®-G_1D_2 (OncoImmunin), was added along with PE-αCD10 and APC-αCD4/CD8. Cells were incubated for an additional 30 min at 37 °C. After treatment, cells were collected, washed, and Raji B-cell ($PE^+/CD4^-/CD8^-$) caspase 3 activation quantified using flow cytometry (FACSCanto). The geometric mean fluorescence of treated cohorts was normalized to untreated control. Experiment was conducted in triplicate and replicated two times. Flow cytometry histogram overlay of PhiPhiLux®-G_1D_2 signal can be found in Supplementary Figure S12.

Apoptosis Assay

Raji B-cells ($1x10^5$) and healthy donor, naïve T-cells ($1x10^5$) were co-cultured in a 1-to-1 B cell-to-T cell ratio in a 48-well plate in 400 µL RPMI 1640 medium. Cells were incubated with or without CD20-directed MATCH (50 nM) or blinatumomab (50 nM) for 4 h at 37 °C. After 4 h, cells were collected, washed and stained with FITC-annexin V, DAPI, PE-αCD10, and APC-αCD4/CD8 for 20 min at 4 °C in annexin V binding buffer. After staining, cells were collected, washed and resuspended in fresh PBS and immediately analyzed by flow cytometry (FACSCAnto) for apoptosis (annexin V^+ and annexin $V^+/DAPI^+$) of Raji B-cells ($CD10^+/CD4^-/CD8^-$). For the FasL blockade assessment, the cells were dosed as described in combination with an anti-FasL antibody. For the granzyme inhibition assessment, healthy donor, naïve T-cells were pre-treated with a protease inhibitor cocktail for 30 min at 37 °C before co-culture with Raji B-cells. The number of apoptotic cells in the FasL blockade and the granzyme inhibitor cohorts were normalized to the respective CD20 MATCH and blinatumomab cohorts to observe amount of apoptosis inhibition for each component. Experiment was performed in triplicate and replicated two times. Gating can be found Supplementary Figure S17.

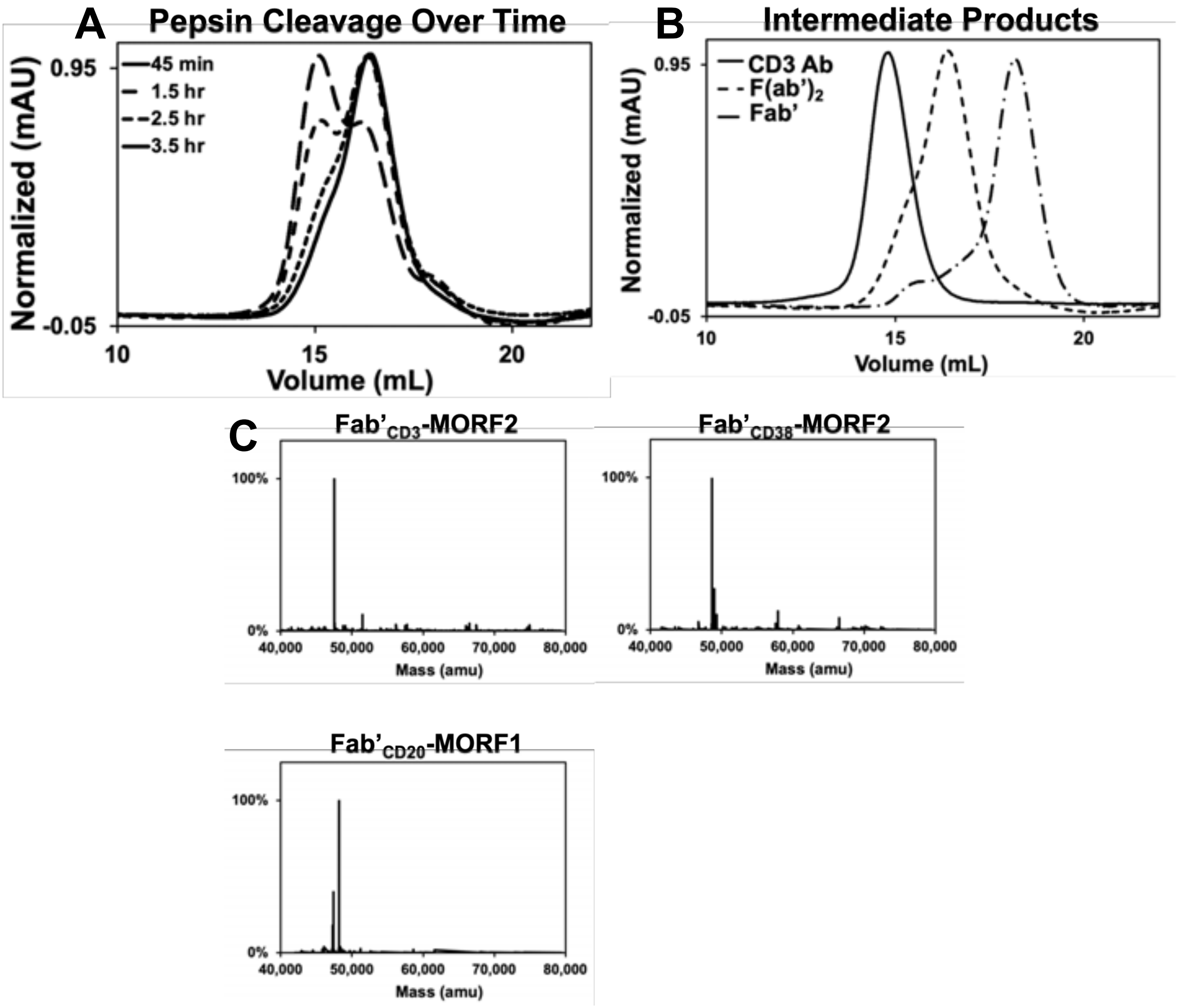

Figure S1. Synthesis characterization of Fab'-MORF MATCH conjugates. **(A)** Time dependence of pepsin cleavage of anti-CD3 antibody monitored by SEC. **(B)** Overlay SEC traces of purified intermediate products of CD3 whole antibody (solid line), $F(ab')_2$ (dotted line), and Fab' species (dot-and-line). Chromatographs were detected on a Superdex 200 10/300 GL column in PBS (pH 7.4) at 0.4 mL min^{-1}. **(C)** Mass spectrometry of Fab'-MORF conjugate final, purified products. Data accompanies Figure 1 of main text.

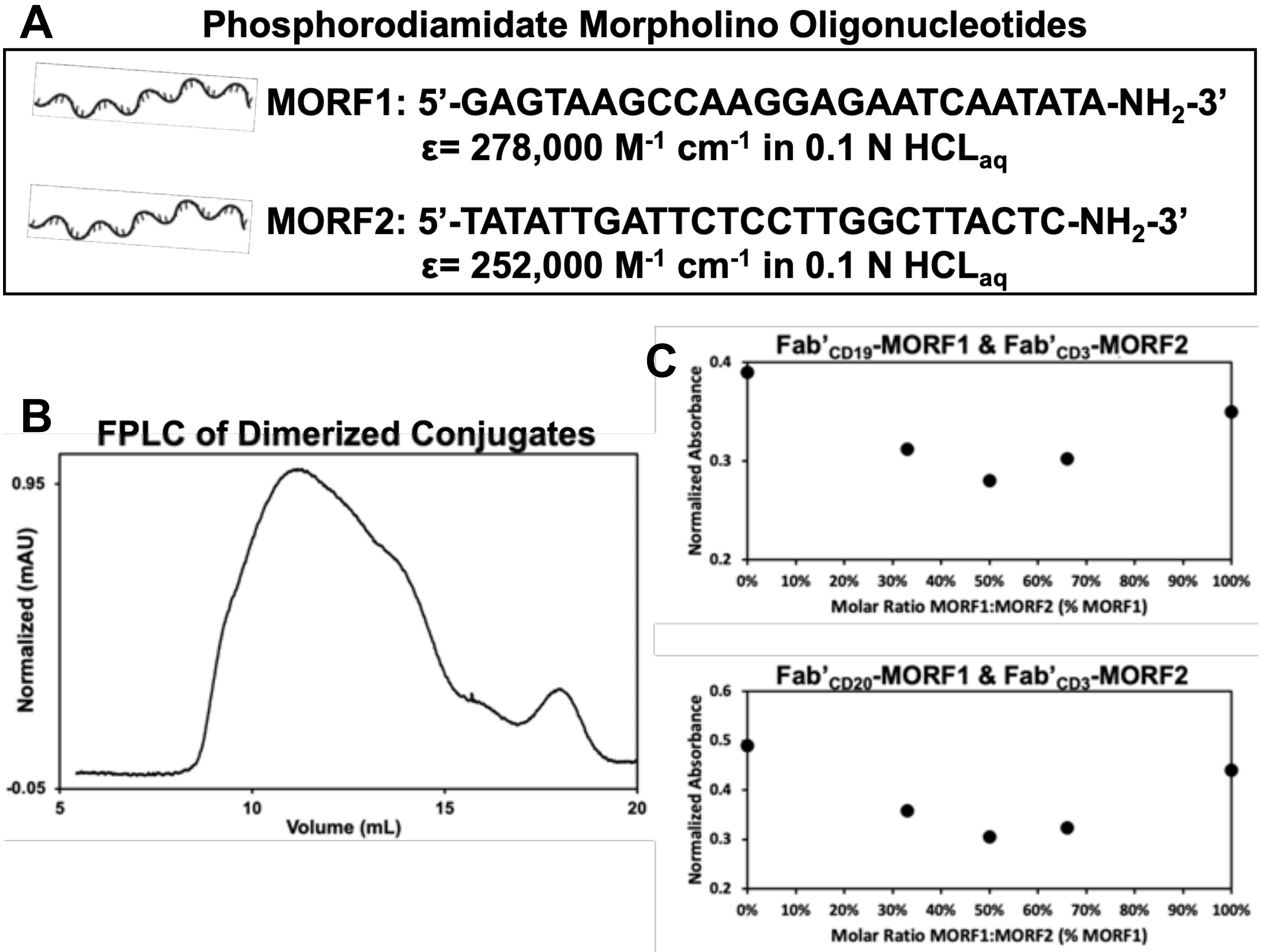

Figure S2. Quality control of synthesized Fab'-MORF conjugates dimerizing via complementary MORF1/MORF2 hybridization. **(A)** Individual MORF sequences. **(B)** SEC chromatogram of premixed Fab'$_{CD20}$-MORF1 with Fab'$_{CD3}$-MORF2. **(C)** UV-Vis absorbance of the hypochromic effect observed at 260 nm when complementary MORF1/MORF2 oligonucleotide strands hybridize. Data accompanies Figure 1 of main text.

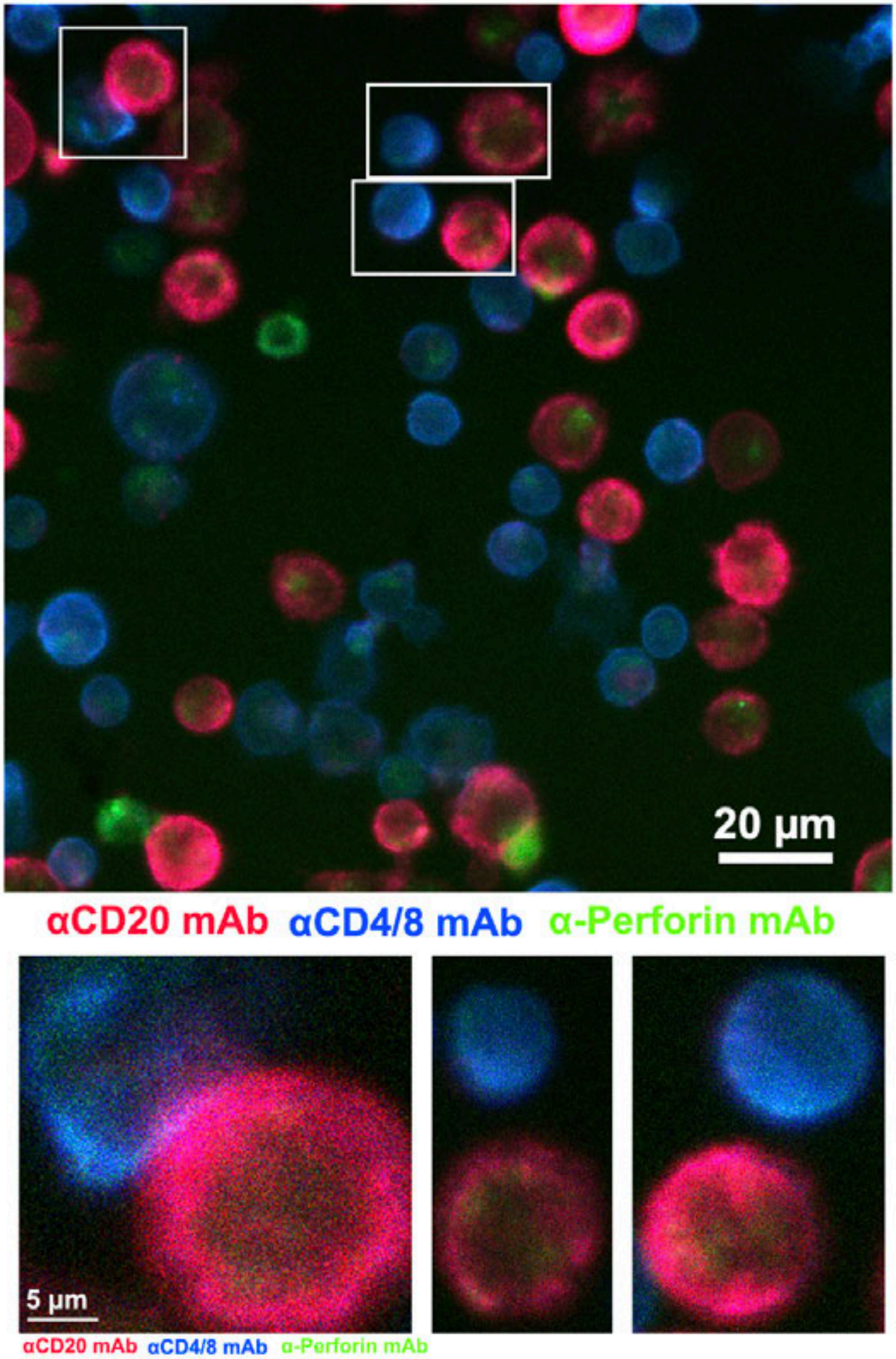

Figure S3. Confocal microscopy of perforin pores formed in cell membrane of target Raji B-cells (red) by T-cells (blue). Figure represents the untreated co-culture control. Data accompanies Figure 2 of main text.

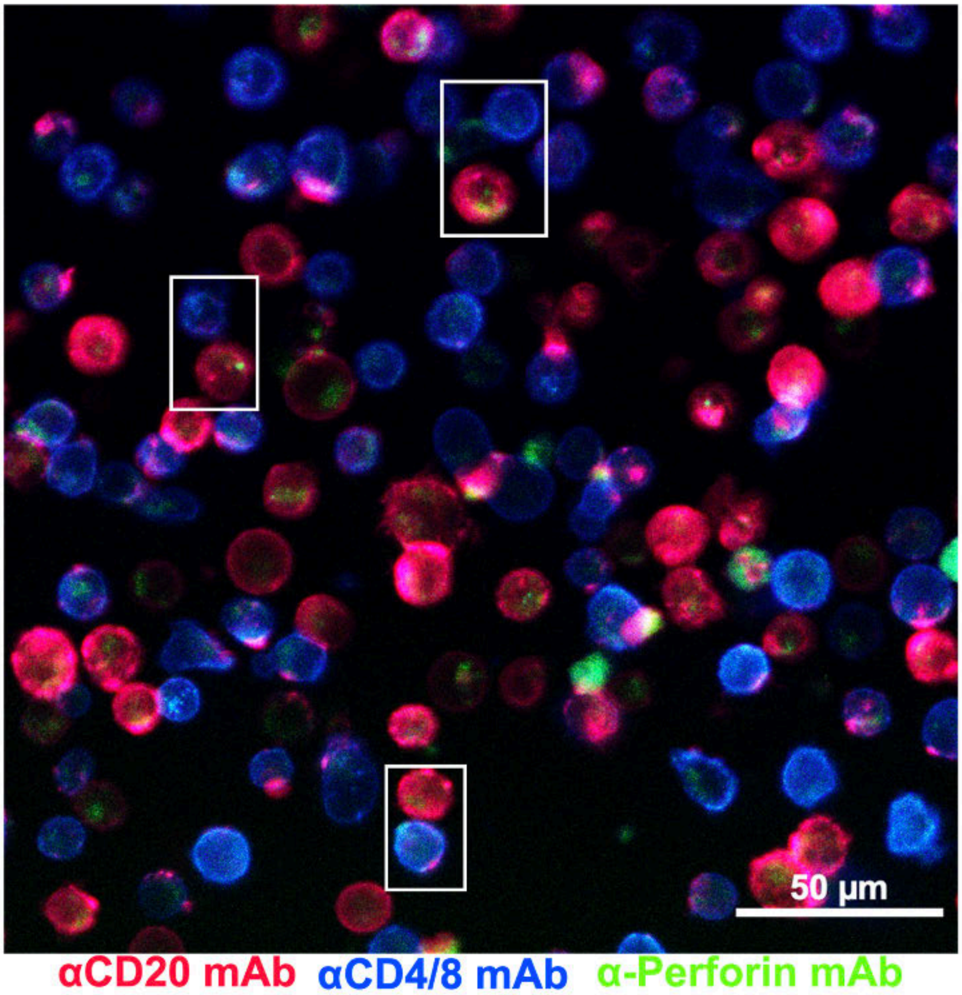

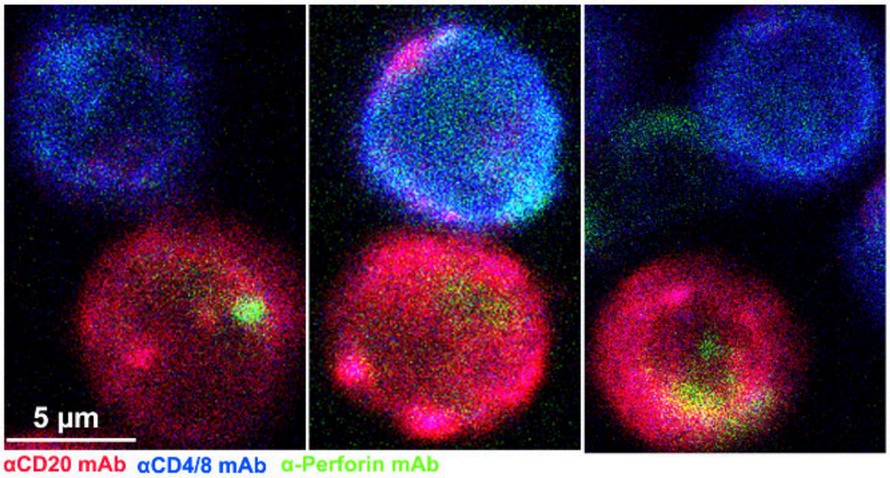

Figure S4. Confocal microscopy of perforin pores (green) formed in cell membrane of target Raji B-cells (red) by T-cells (blue). Figure represents the blinatumomab-treated co-culture. Data accompanies Figure 2 of main text.

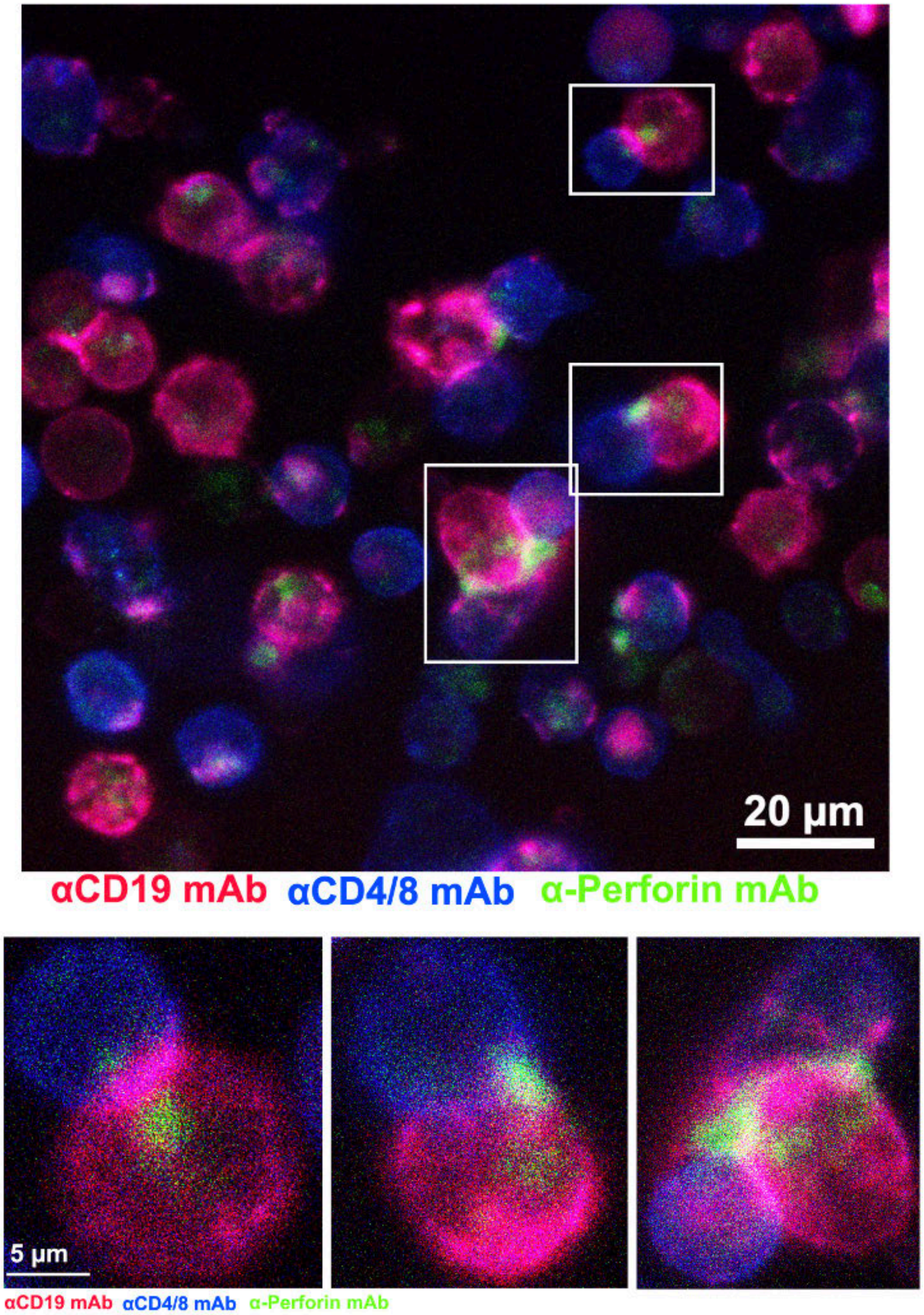

Figure S5. Confocal microscopy of perforin pores (green) formed in cell membrane of target Raji B-cells (red) by T-cells (blue). Figure represents the CD20 MATCH-treated co-culture. Data accompanies Figure 2 of main text.

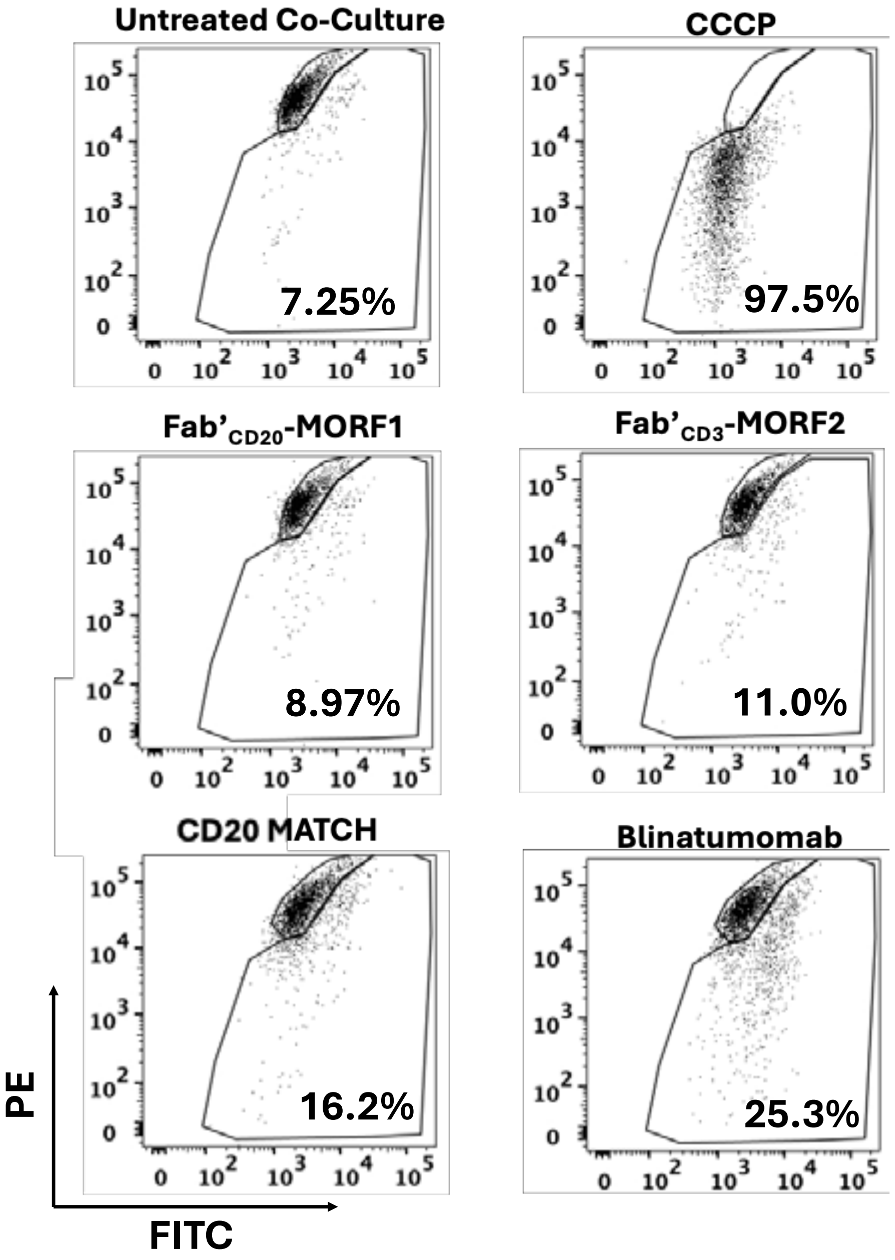

Figure S6. Flow cytometry gating of mitochondrial depolarization. Specifically gating JC-1 aggregation (FITC) and JC-1 monomers (FITC). Healthy cells should be PE$^+$ and FITC-*dim*. Data accompanies Figure 2 of main text.

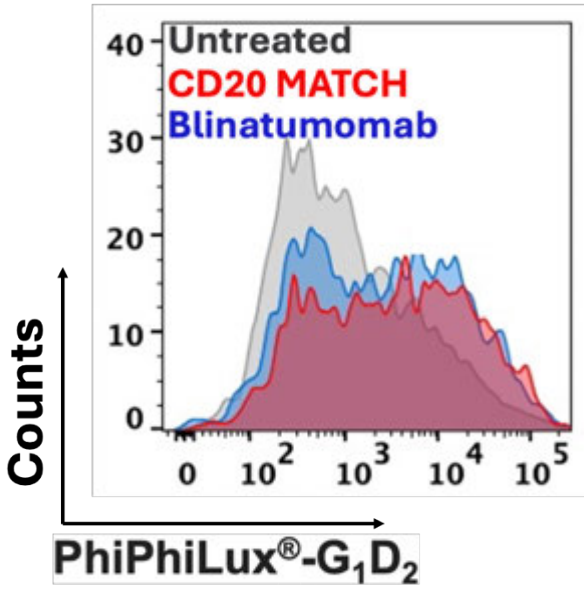

Figure S7. Overlayed flow cytometry histograms of caspase-3 activation. Data accompanies Figure 2 of main text.

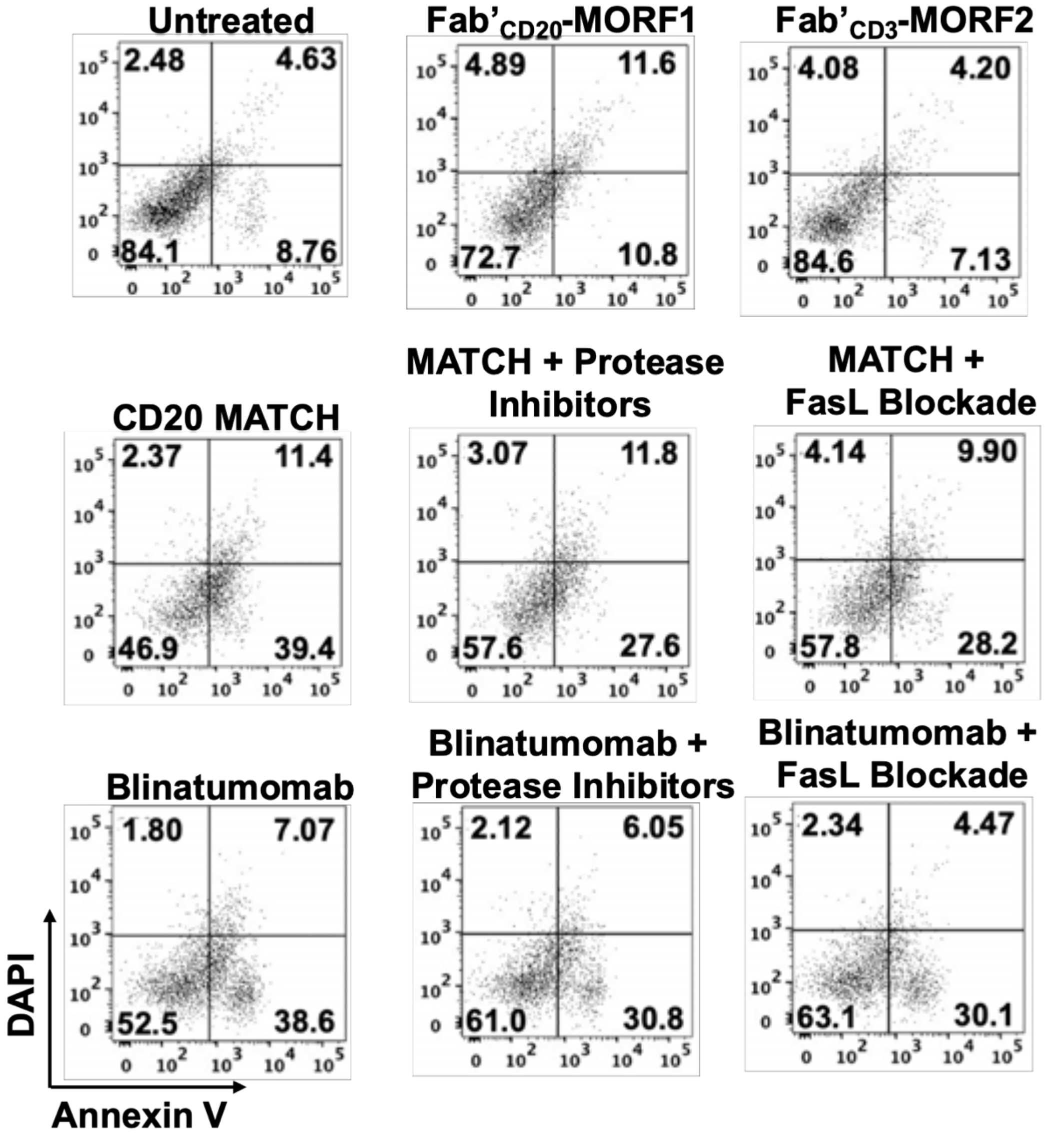

Figure S8. Apoptosis quantification gating of MATCH with or without protease inhibitor or FasL blockade. Data accompanies Figure 3 of main text. Percentage of cells in quadrants 1, 2 and 4 ($DAPI^+$/annexin V^-, $DAPI^+$/annexin V^+, and $DAPI^-$/annexin V^+) were summed and normalized to untreated control cells and presented as normalized apoptotic cells. Data accompanies Figure 2 of main text.

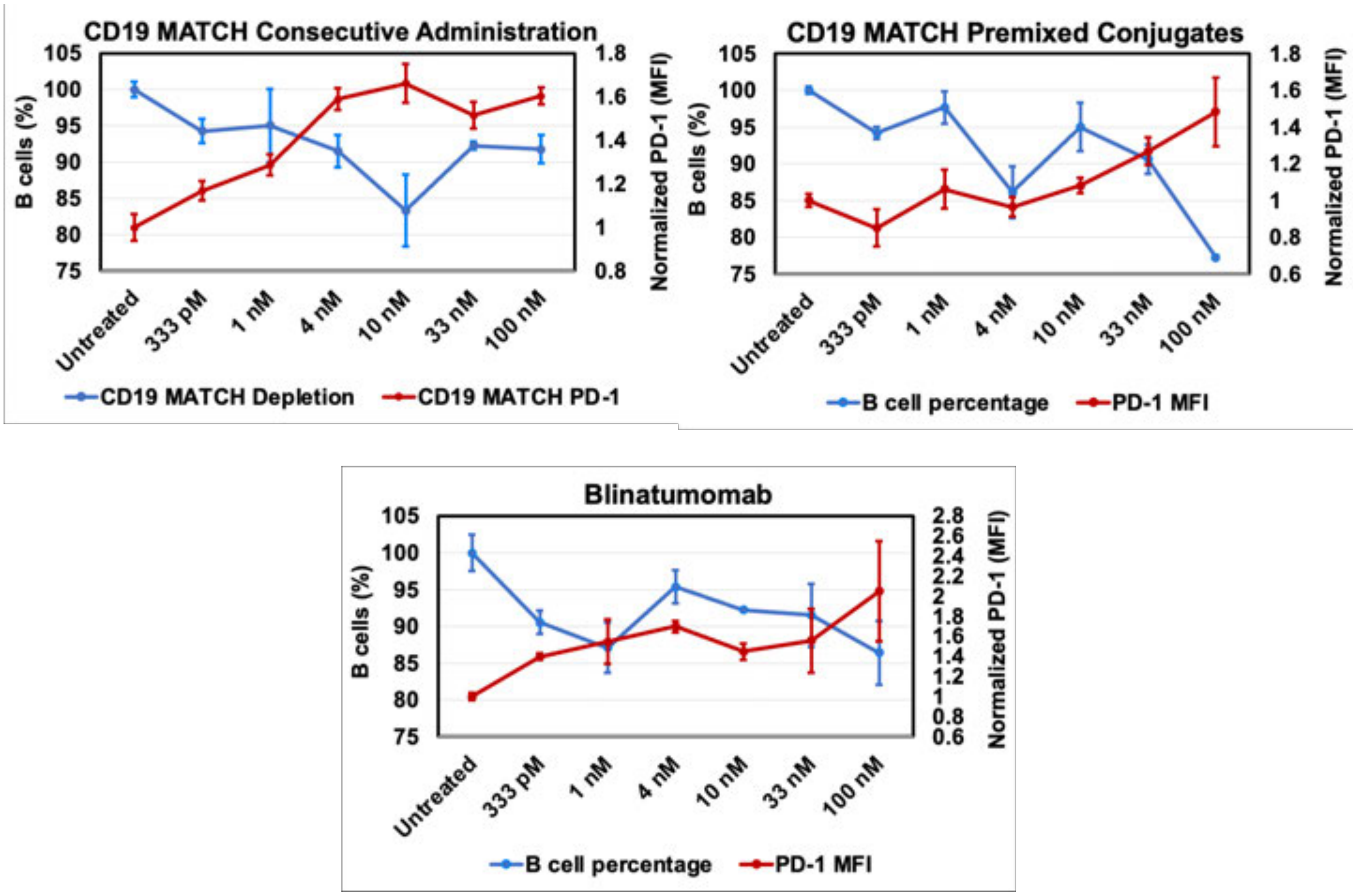

Figure S9. Consecutively dosed CD19-directed MATCH (left) versus premixed dosed CD19-directed MATCH (right) compared to blinatumomab (bottom). Red lines represent percentage of B-cell depletion after 24 treatment. Blue lines represent levels of PD-1 expression on cytotoxic T-cell upon activation with the therapeutic. Data accompanies Figure 3 of main text.

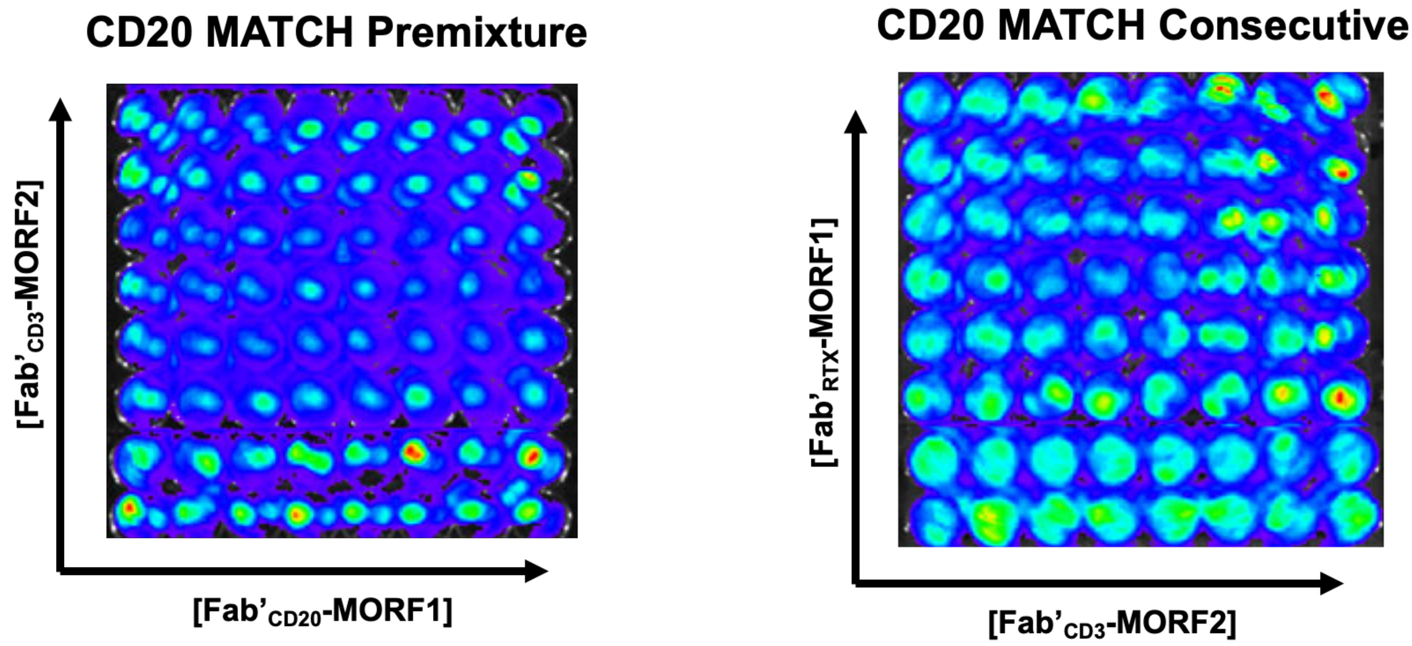

Figure S10. Original bioluminescence of luciferase-expressing Raji cells remaining in a 48-well plate after 24 h incubation with CD20-directed MATCH administered as either a premixed dose (left) or consecutively (right). Data accompanies Figure 3 of main text.

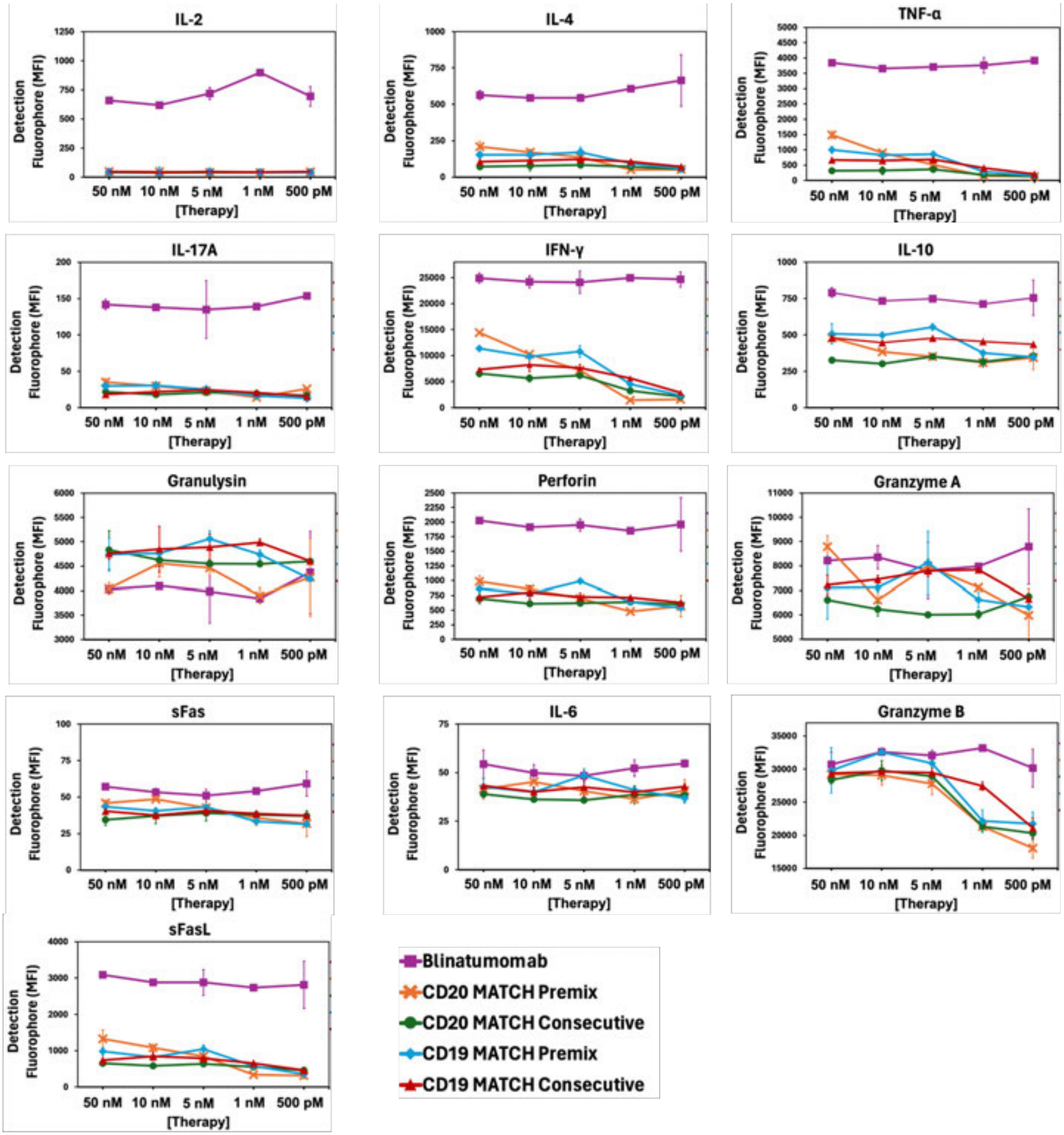

Figure S11. Full *in vitro* T-cell cytokine quantification data acquired using a multiplex kit. Data accompanies Figure 3 of main text.

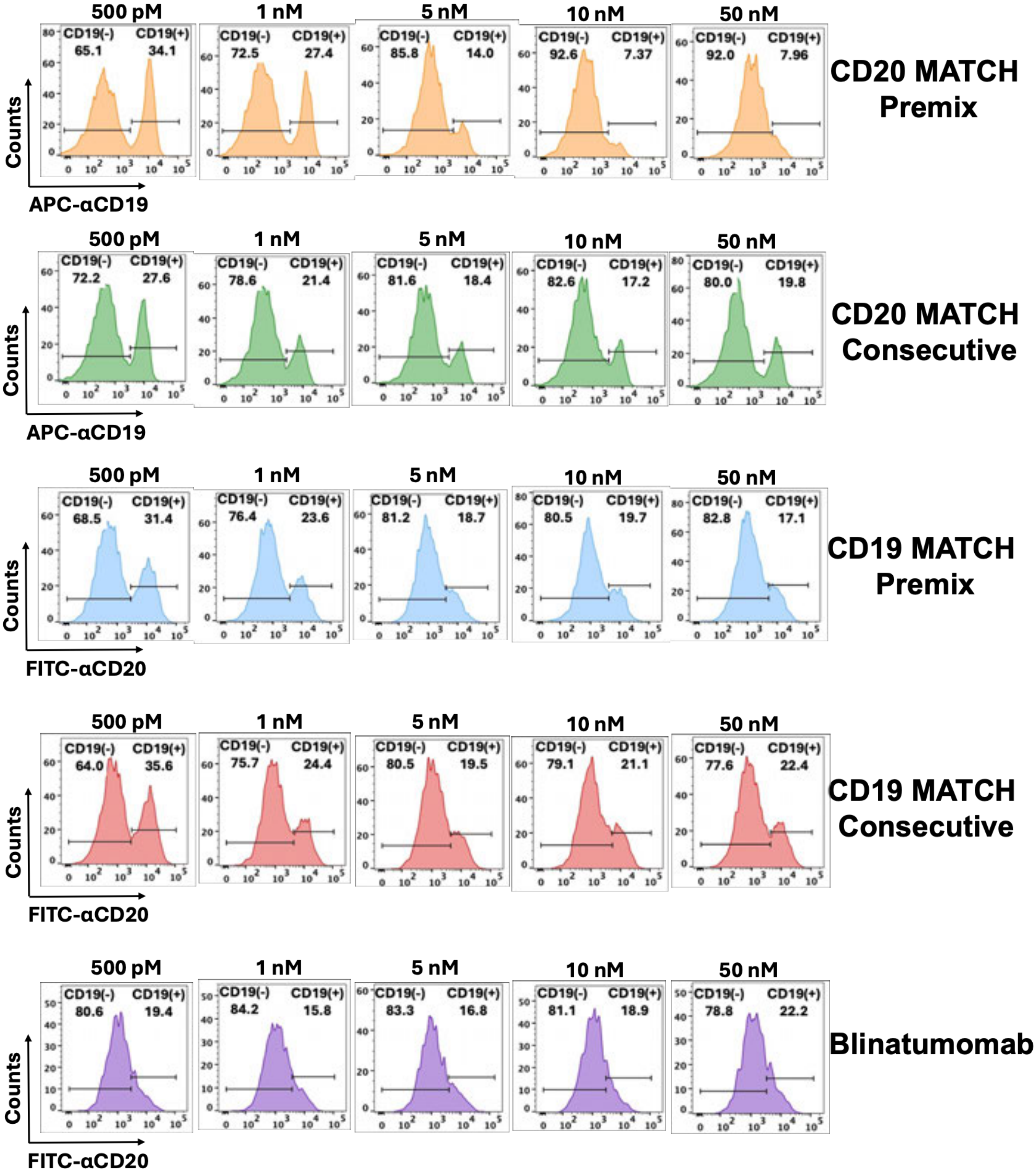

Figure S12. Flow histograms of residual Raji B-cells after 24 h incubation with indicated therapy. Data accompanies Figure 3 of main text.

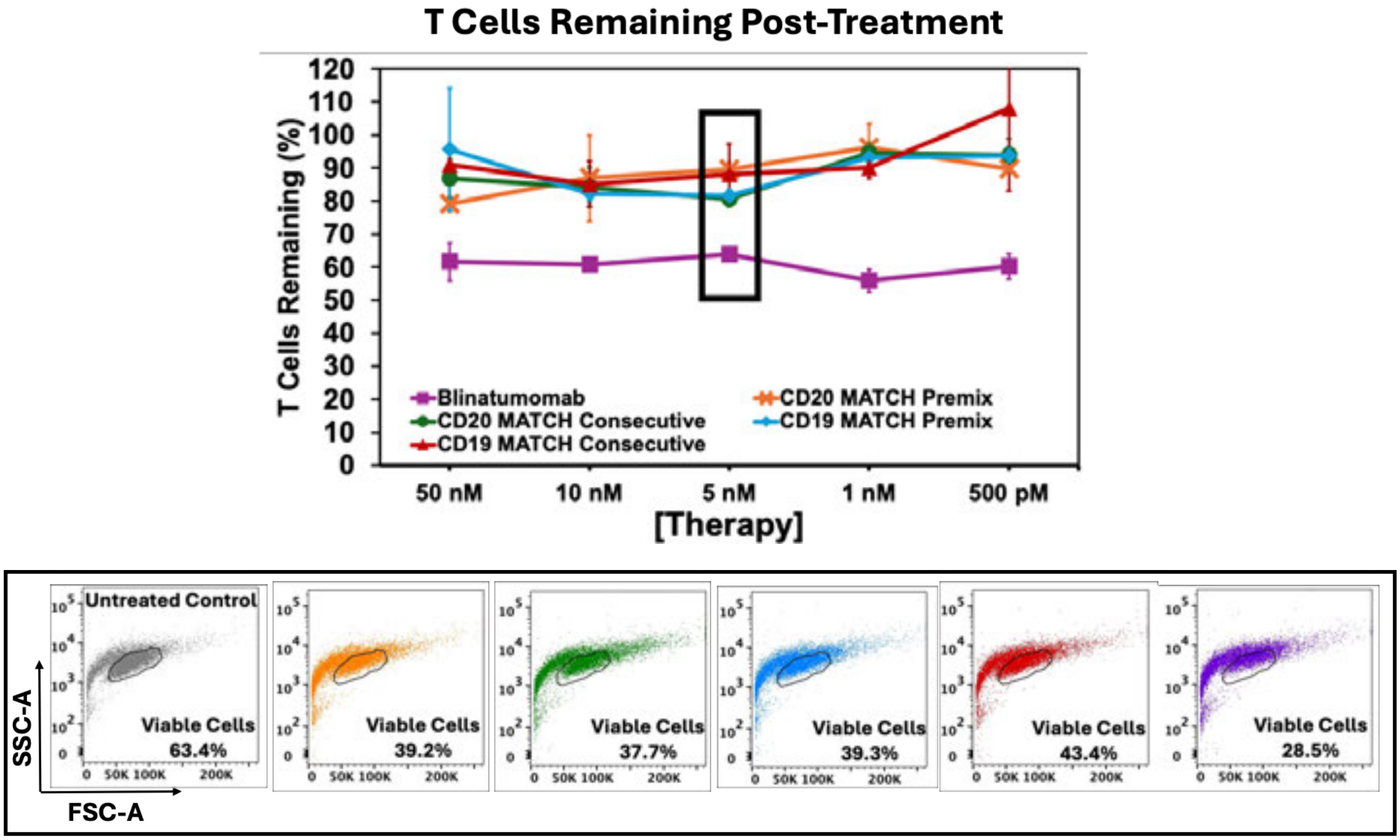

Figure S13. T-cell viability after 24 h incubation with indicated therapy. Gating shows number of viable T-cells and Raji B-cells. T-cell counts were quantified using CD3 immunostaining and flow cytometry. Data accompanies Figure 3 of main text.

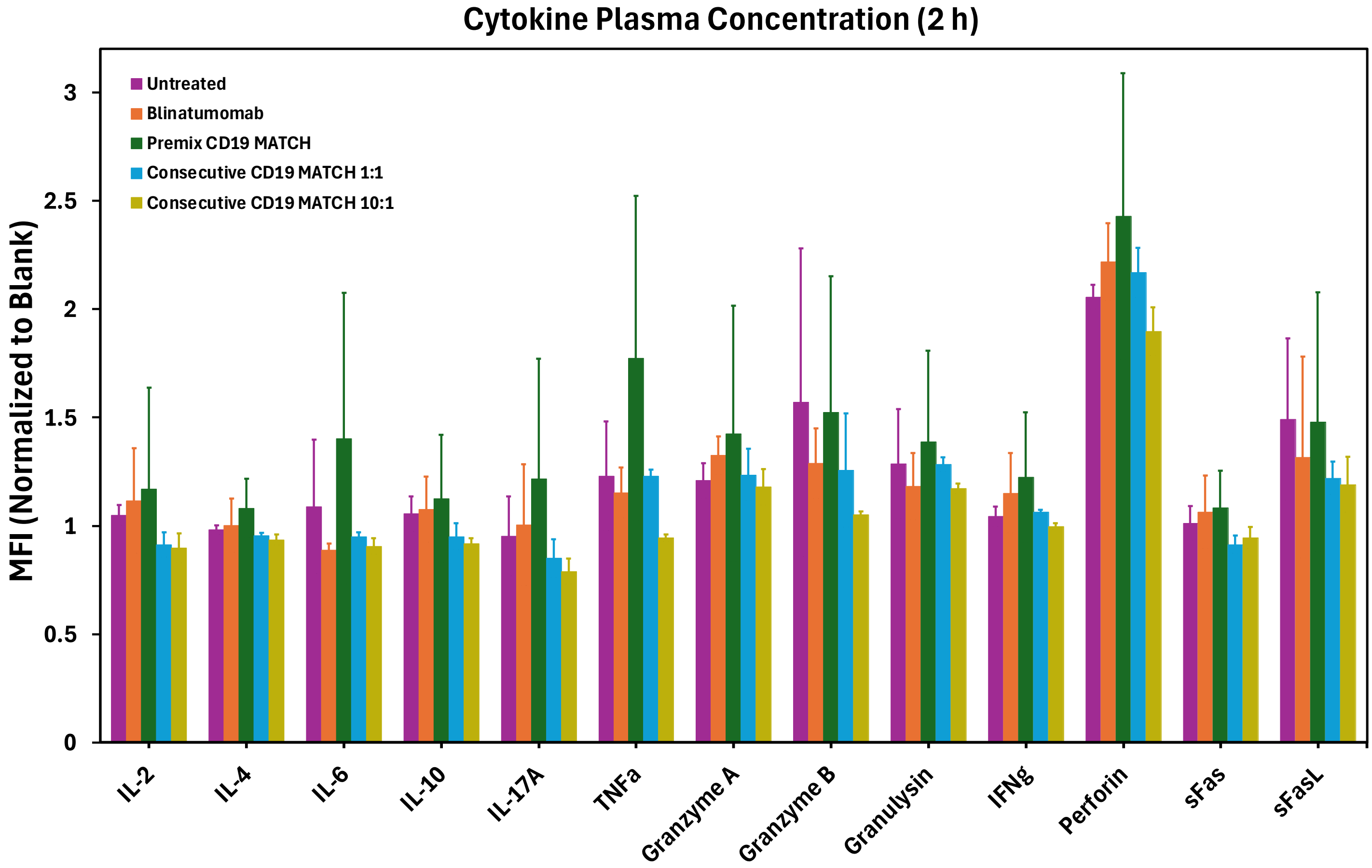

Figure S14. *In vivo* plasma concentrations of cytokines and interleukins after treatment with MATCH or blinatumomab (2 h). Data was quantified using a multiplex kit and flow cytometry and is presented as the mean MFI for each group, normalized to plasma collected from a cohort without treatment or injected healthy human T cells (n=3 for each cohort). Data accompanies Figure 3 of main text.

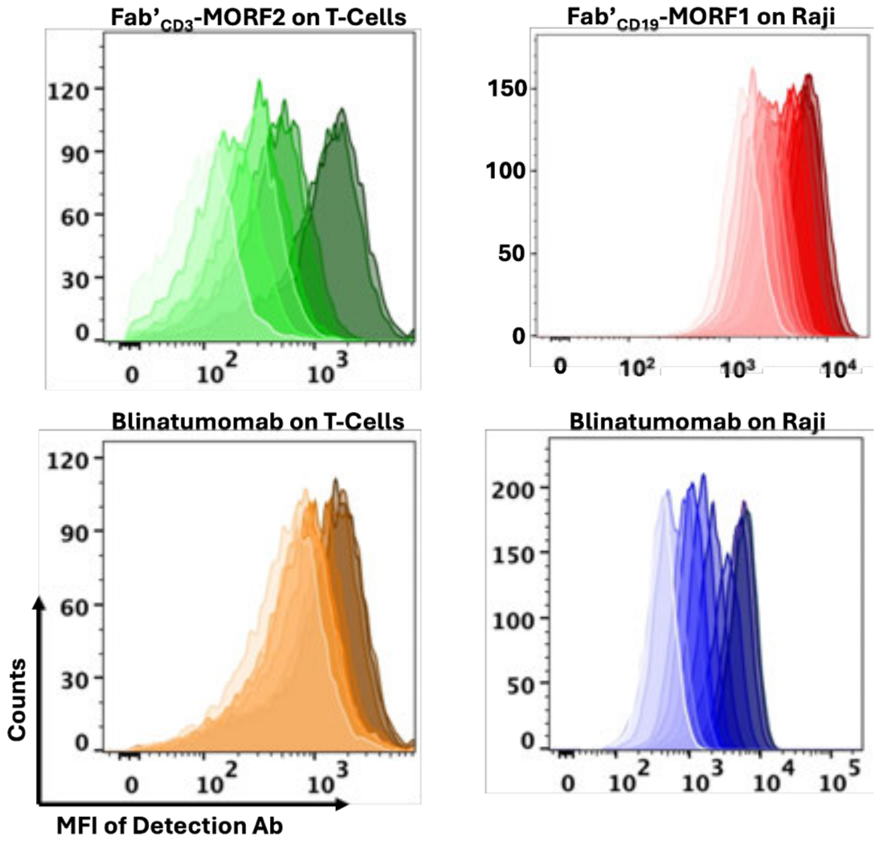

Figure S15. Flow cytometry histograms of competitive binding assay to compare binding of blinatumomab to Fab'$_{CD19}$-MORF1 and Fab'$_{CD3}$-MORF2 conjugates on the surface of Raji B-cells and T-cells. Data accompanies Figure 3 of main text.

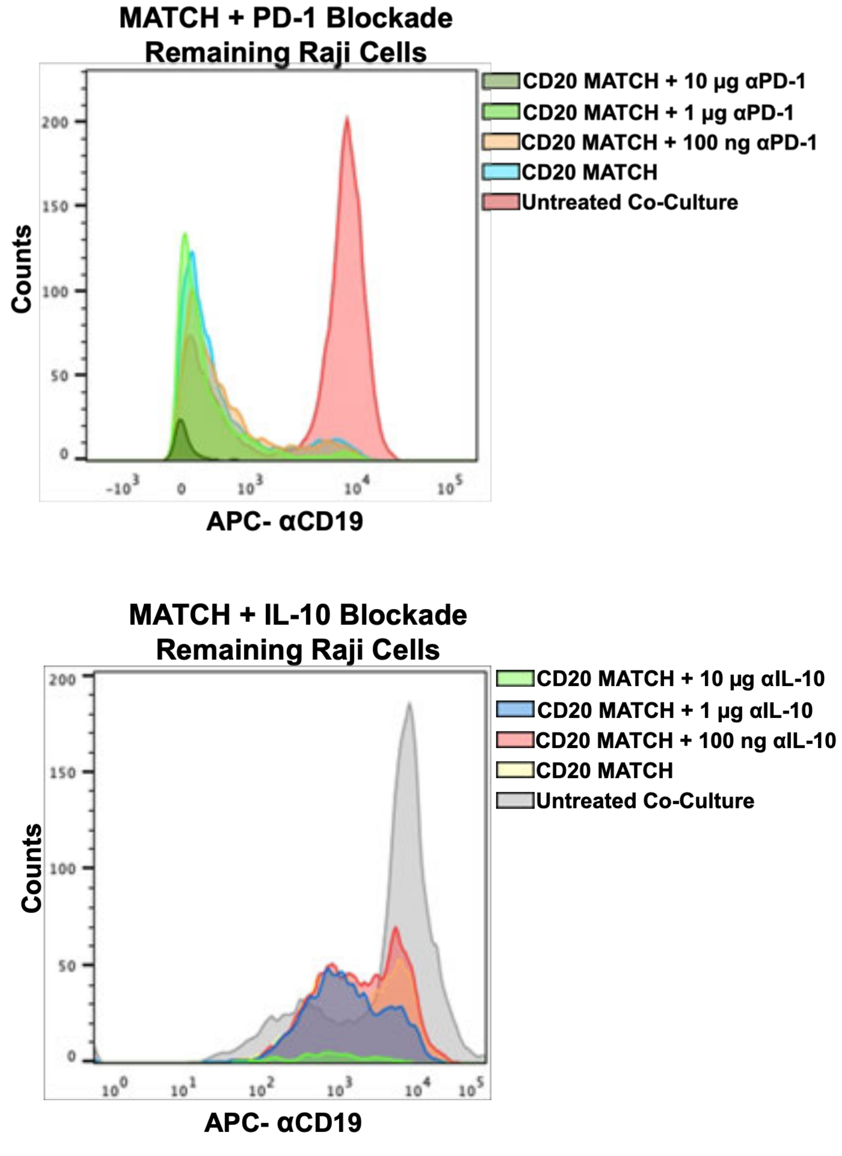

Figure S16. Overlayed MFIs of CD19(+) Raji cells when dosed with CD20-directed MATCH administered in combination with either a PD-1 blockade (top) or IL-10 blockade (bottom). Histograms correspond to Figure 3 of the main text.

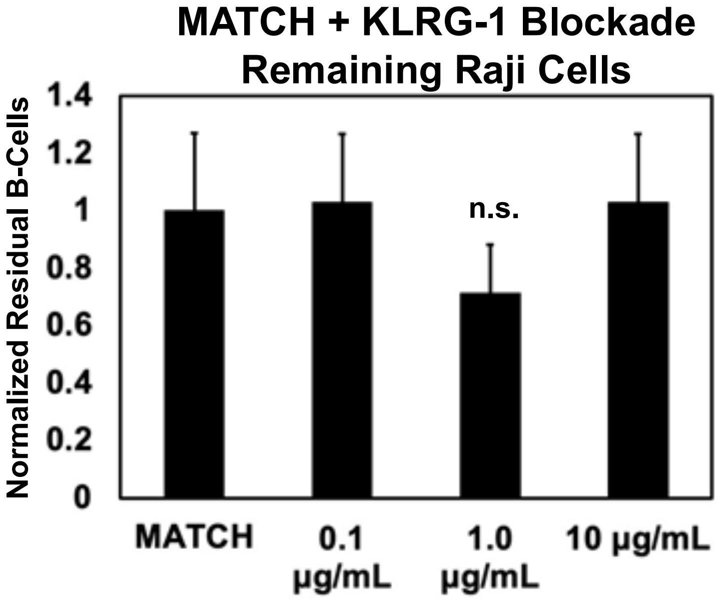

Figure S17. Residual target cells in CD20-directed MATCH, with or without, anti-KLRG-1 antibody combination therapy (normalized to MATCH-treated co-culture). No significant improvement to MATCH-induced cell killing was observed at any dose combination tested.

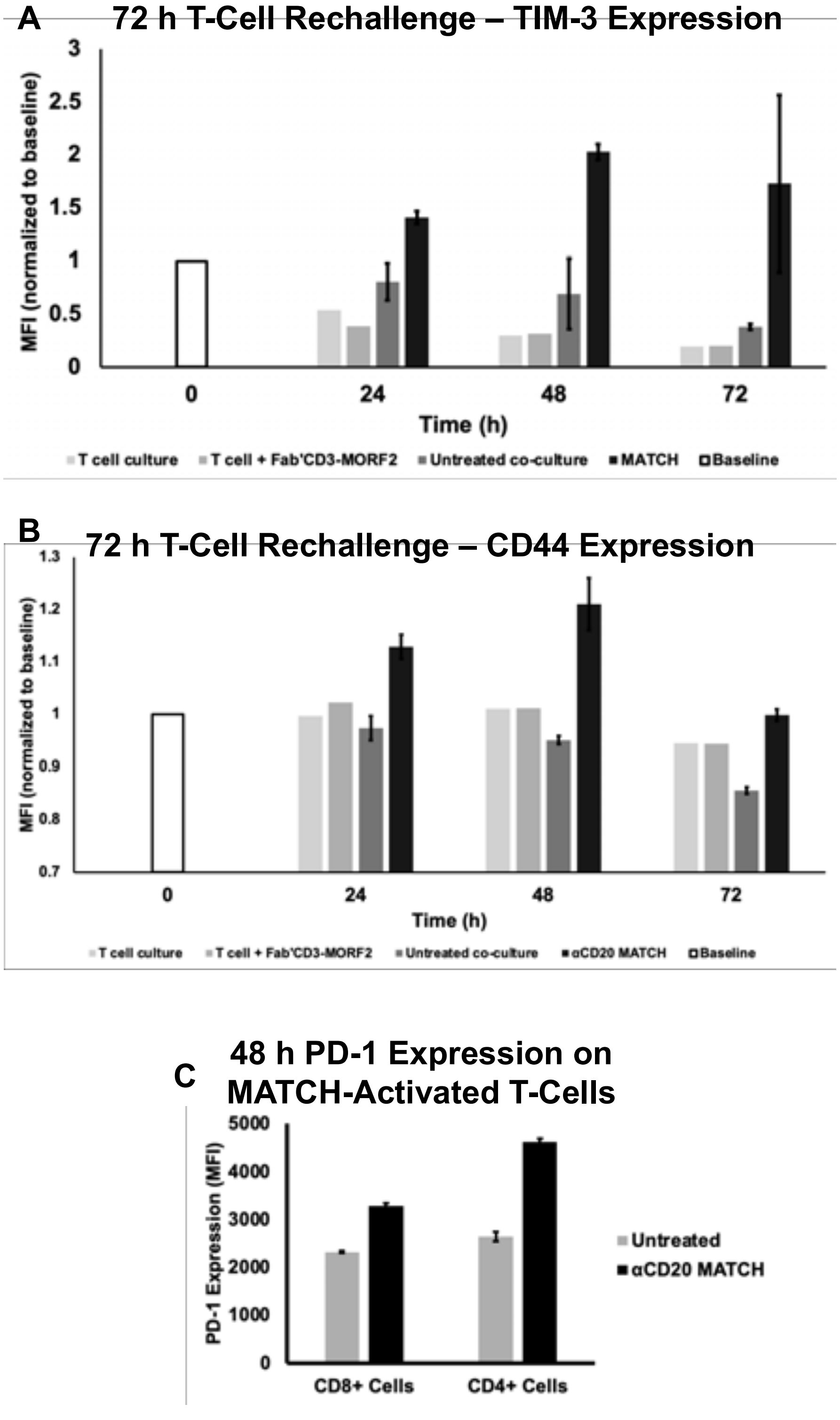

Figure S18. T-cell activation and exhaustion markers of T-cell being rechallenge with multiple rounds of target B-cells. **(A)** TIM-3 expression on T-cells that were rechallenged with Raji cells every 24 h for 72 h. **(B)** CD44 expression on T-cells that were rechallenged with Raji cells every 24 h for 72 h. **(C)** PD-1 expression on T-cells rechallenged with Raji cells every 24 h for 48 h. T-cells were able to kill multiple rounds of target cells with a fresh dose of CD20-directed MATCH every 24 h.

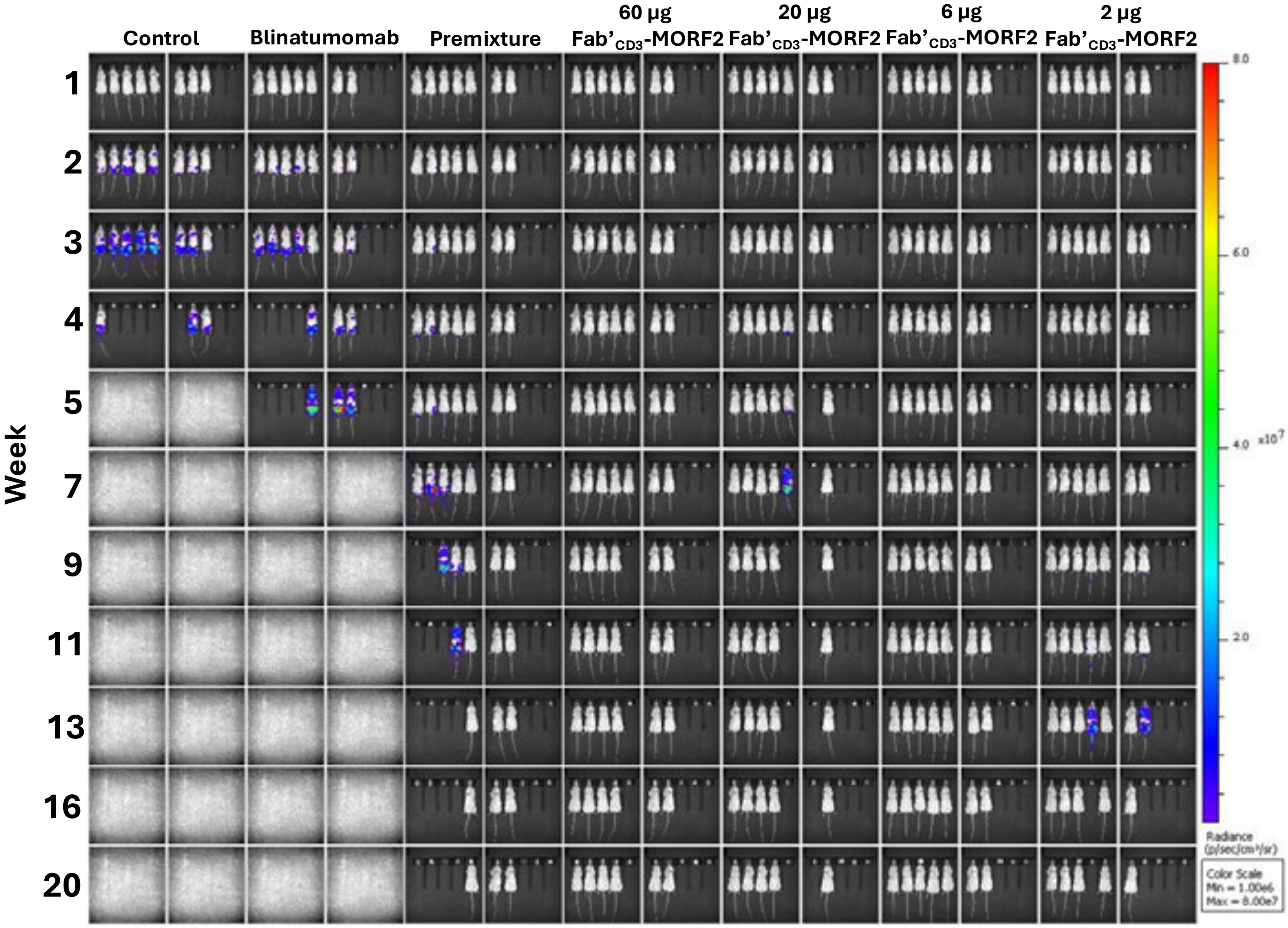

Figure S19. Complete IVIS imaging for *in vivo* assessment of CD20-directed MATCH. Data accompanies Figure 4 of main text.

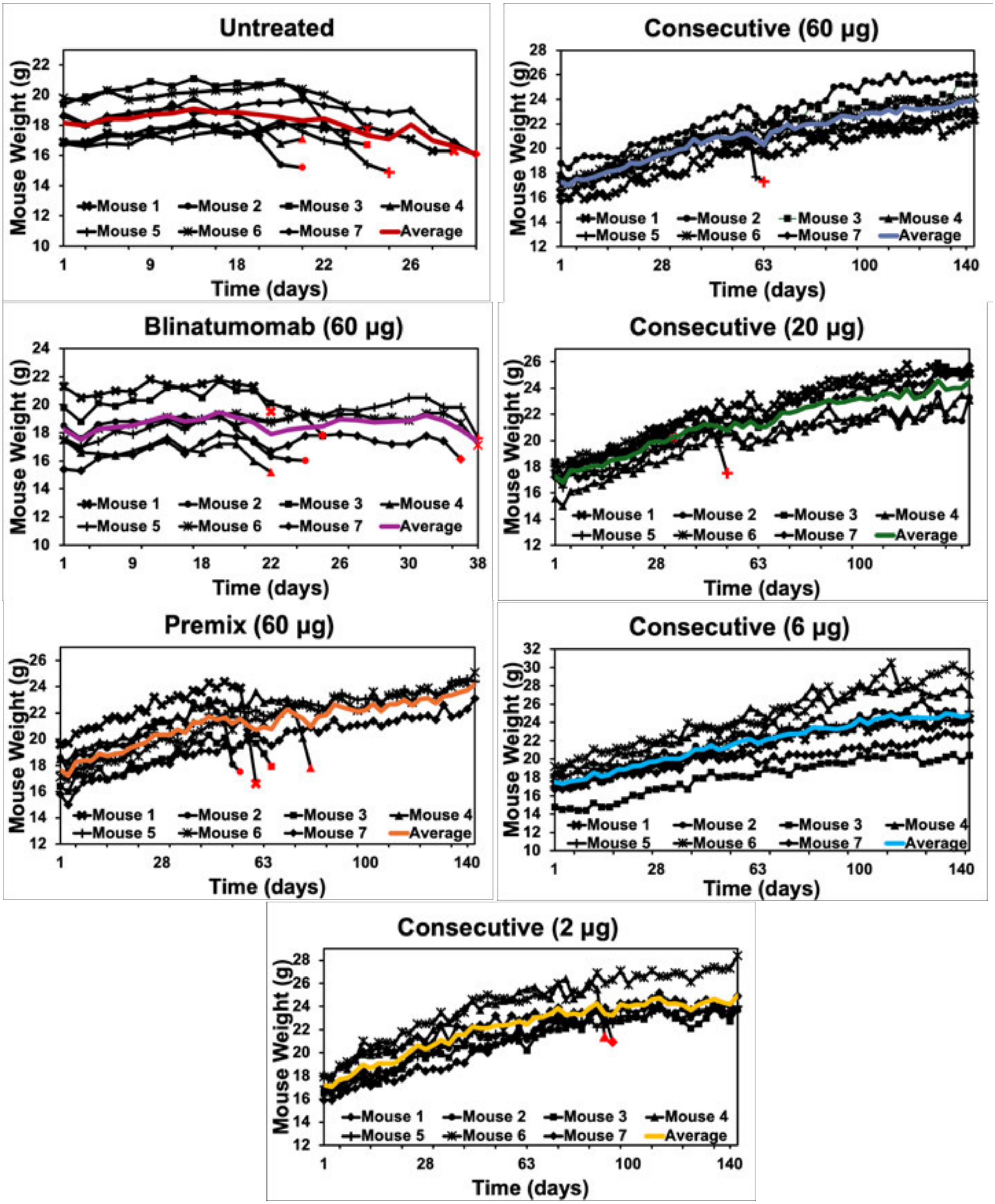

Figure S20. Individual mouse weights for *in vivo* assessment of CD20-directed MATCH. Data accompanies Figure 4 of main text.

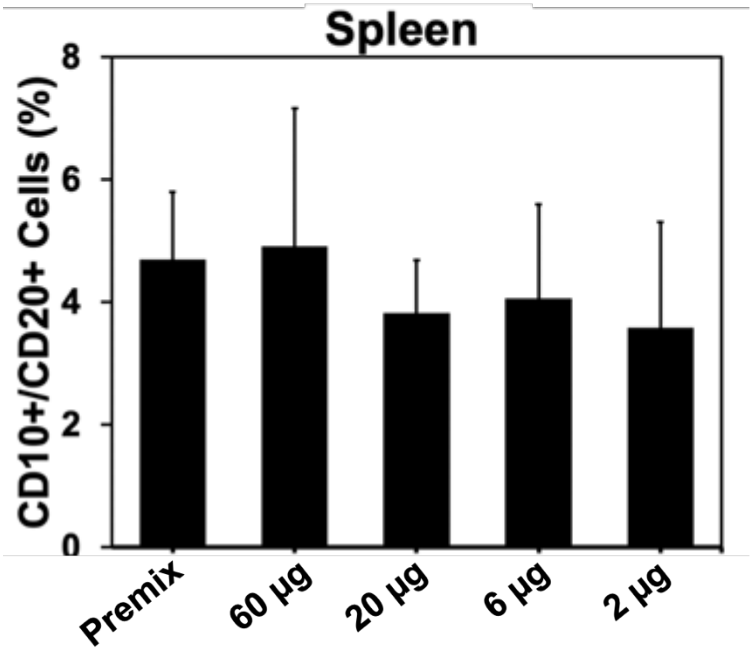

Figure S21. *Ex vivo* residual Raji B-cells (CD10^{+}/CD20^{+}) in mouse spleen for *in vivo* assessment of CD20-directed MATCH. Data accompanies Figure 4 of main text.

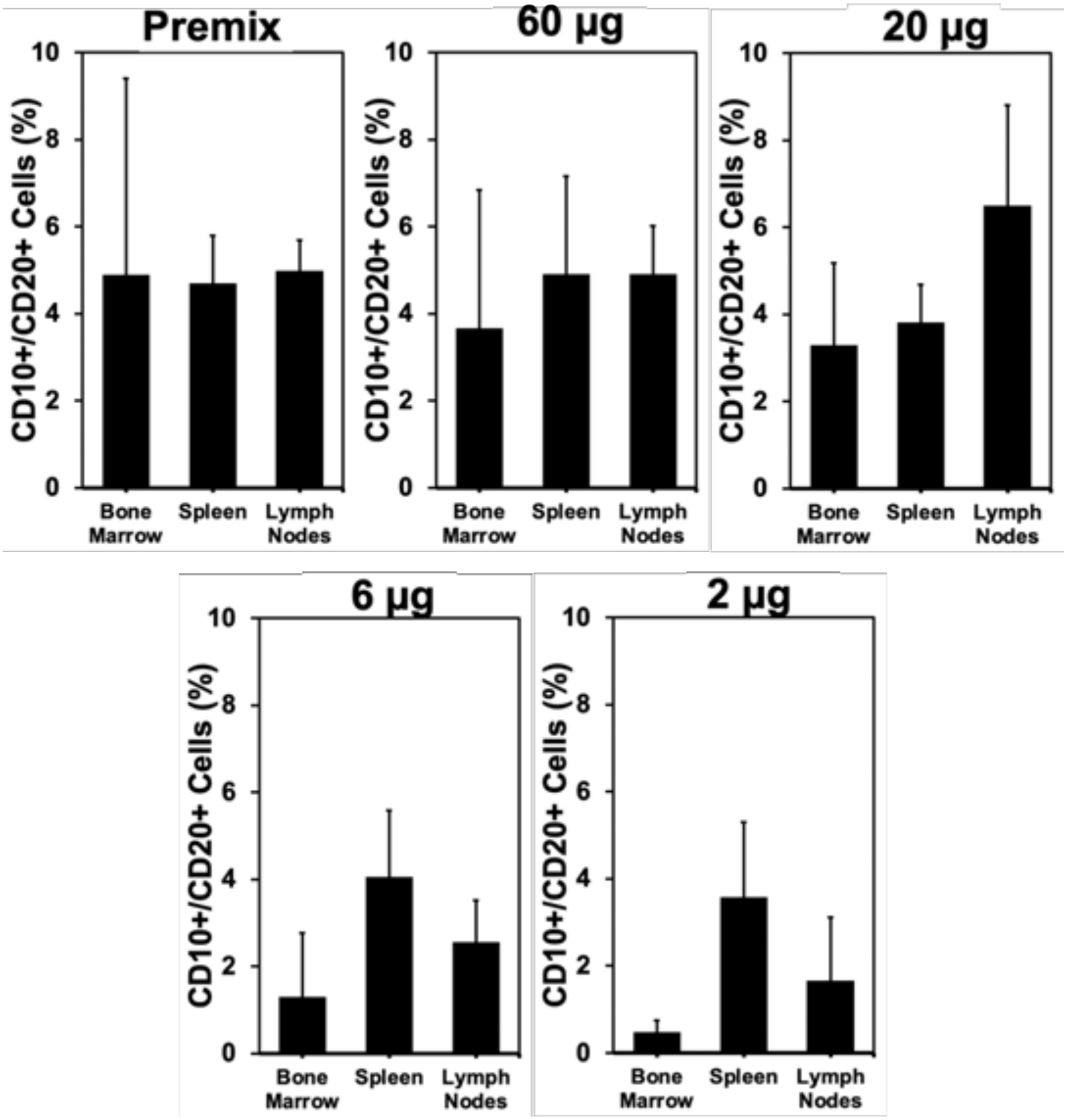

Figure S22. *Ex vivo* residual Raji B-cells ($CD10^{+}/CD20^{+}$) for *in vivo* assessment of CD20-directed MATCH – group tissue comparison. Data accompanies Figure 4 of main text.

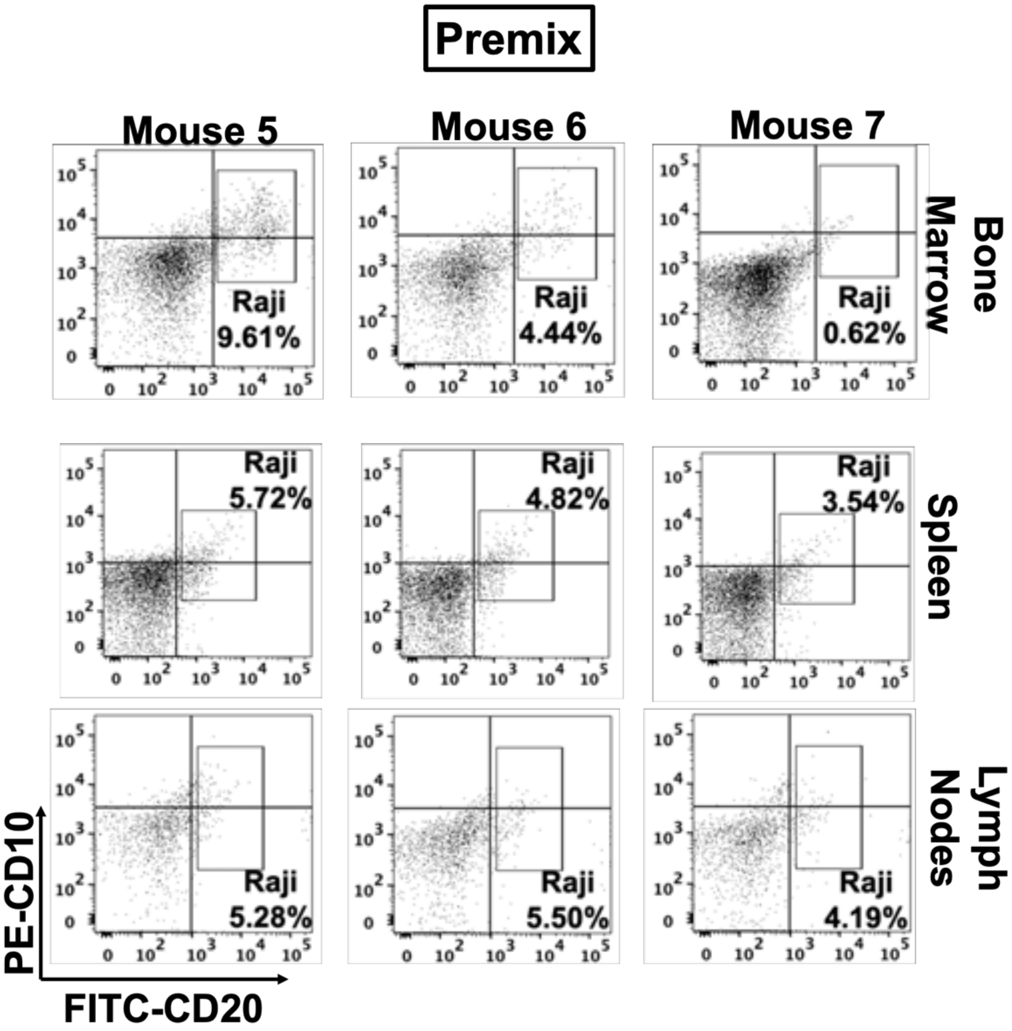

Figure S23. Flow cytometry residual disease quantification for premixed-treated cohort's long-term survivors. Data accompanies Figure 4 of main text.

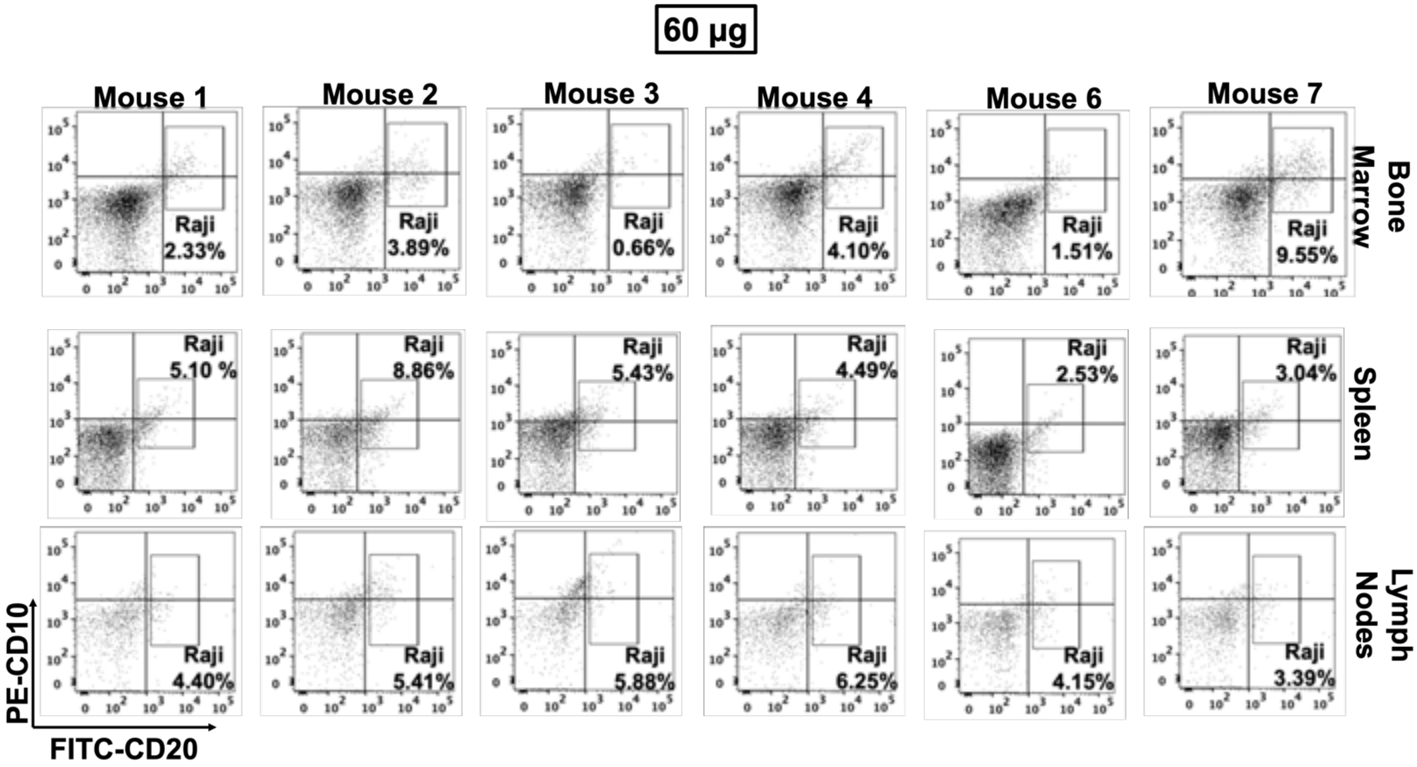

Figure S24. Flow cytometry residual disease quantification for the 60 µg T-cell engager-treated cohort's long-term survivors. Data accompanies Figure 4 of main text.

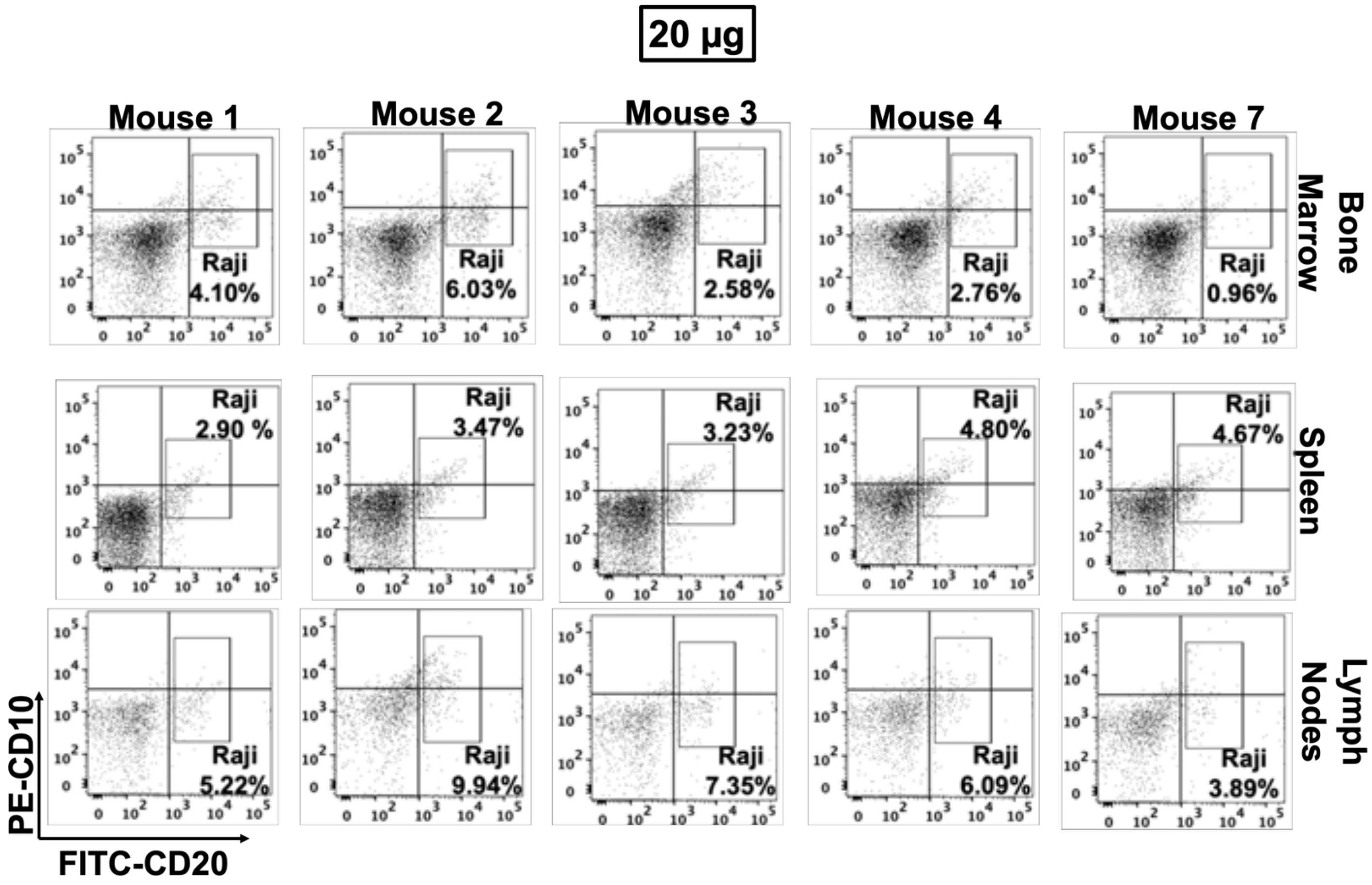

Figure S25. Flow cytometry residual disease quantification for the 20 μg T-cell engager-treated cohort's long-term survivors. Data accompanies Figure 4 of main text.

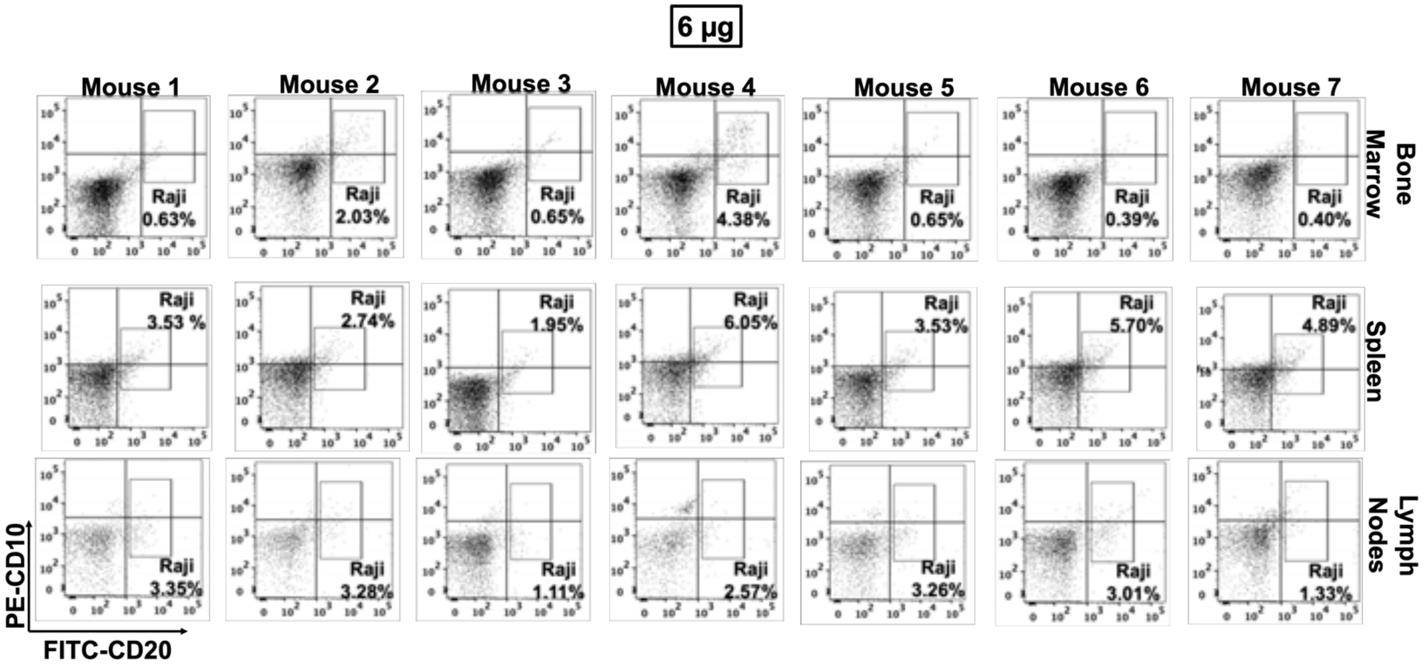

Figure S26. Flow cytometry residual disease quantification for the 6 µg T-cell engager-treated cohort's long-term survivors. Data accompanies Figure 4 of main text.

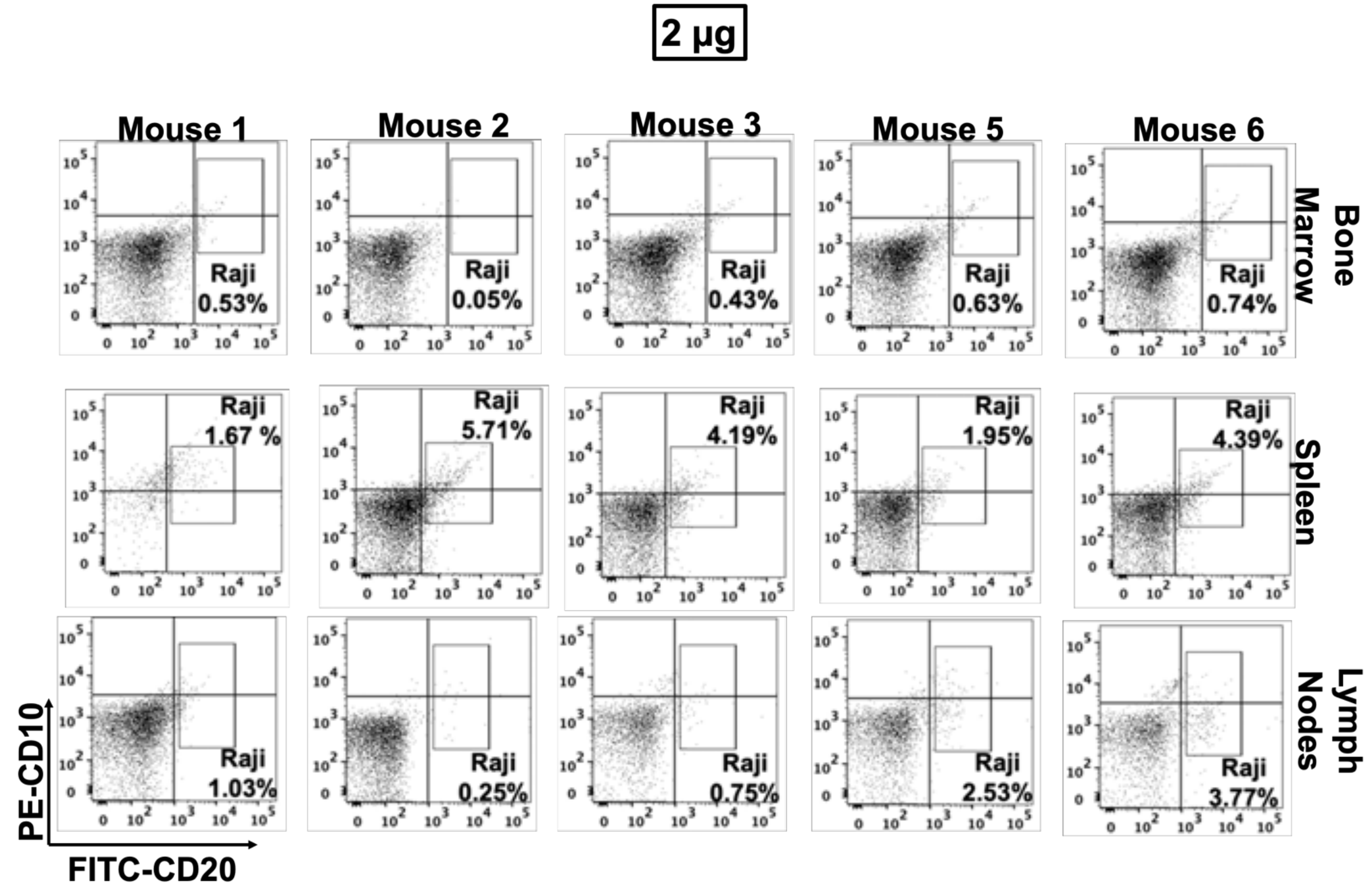

Figure S27. Flow cytometry residual disease quantification for the 2 µg T-cell engager-treated cohort's long-term survivors. Data accompanies Figure 4 of main text.
